# Bulk and Single-Cell Transcriptome Analyses Revealed That the Pyroptosis of Glioma-Associated Macrophages Participates in Tumor Progression and Immunosuppression

**DOI:** 10.1155/2022/1803544

**Published:** 2022-09-26

**Authors:** Lin Li, Leyang Wu, Xingpeng Yin, Chenyang Li, Zichun Hua

**Affiliations:** ^1^The State Key Laboratory of Pharmaceutical Biotechnology, School of Life Sciences, Nanjing University, Nanjing, China; ^2^Changzhou High-Tech Research Institute of Nanjing University and Jiangsu Target Pharma Laboratories Inc., Changzhou, China; ^3^School of Biopharmacy, China Pharmaceutical University, Nanjing, China

## Abstract

Glioma is the most common of all central nervous system (CNS) malignancies and is associated with a poor prognosis. Pyroptosis has been proven to be associated with the progression of multiple tumors and CNS diseases. However, the relationships between pyroptosis and clinical prognosis and immune cell infiltration are unclear in glioma. In this study, we conducted a comprehensive exploration of pyroptosis in glioma. First, prognosis-related genes were screened at each key regulatory locus in the pyroptosis pathway, and the prognostic ability and coexpression relationships of GSDMD and its upstream pathway genes NLRC4/CASP1/CASP4 were identified and well validated in multiple datasets. Tissue microarray-based immunohistochemistry results showed higher levels of NLRC4 and N-terminal GSDMD in high-grade gliomas, providing conclusive evidence of pyroptosis in gliomas. The robustness of the prognostic model based on these four genes was well validated in TCGA and CGGA cohorts. Bulk RNA-seq-based analysis showed that the group defined as the high-risk group according to the model showed activation of multiple inflammatory response pathways and impaired synaptic gene expression and had a higher infiltration of bone marrow-derived macrophages (BMDMs) and a hypersuppressed immune microenvironment. More importantly, three independent single-cell RNA-seq (scRNA-seq) datasets demonstrated that tumor-infiltrating macrophages, particularly BMDMs but not tissue-resident microglia, showed significant coexpression of the GSDMD and CASP genes, and BMDMs from high-grade gliomas accounted for a higher proportion of immune infiltrating cells and had higher expression of pyroptosis genes. Finally, we revealed the activation of pathways in response to LPS/bacteria and oxidative stress during BMDM development toward the pyroptosis cell fate by pseudotime trajectory analysis, suggesting potential BMDM pyroptosis initiators. The above results provide not only novel insights into the pathological mechanisms of glioma but also novel therapeutic targets for glioma, suggesting the potential application of pyroptosis inhibitors (e.g., disulfiram).

## 1. Introduction

Gliomas, which usually originate from glial cells or precursor cells and progress to astrocytomas, oligodendrogliomas, ventricular meningiomas, or oligodendroglial astrocytomas, account for approximately 80% of malignant tumors of the central nervous system (CNS) [1]. The World Health Organization classifies gliomas into 4 grades [2]. The 10-year survival rate for low-grade gliomas (grades I-II) is 47%, and the median survival time is 11.6 years, while the median overall survival for grade IV gliomas is worse, at 15 months. With advances in targeted tumor treatment research and technology, there have been several breakthroughs in the identification of glioma molecular markers, such as isocitrate dehydrogenase (IDH) mutations [3] and O6-methylguanine-DNA methyltransferase (MGMT O6) promoter methylation [4]. However, these established markers are limited in their ability to elucidate the pathogenesis of glioma and are difficult to translate into targeted therapeutics. Therefore, it is urgent to explore new diagnostic assessment and prognostic analysis strategies for glioma pathogenesis and progression mechanisms and to obtain novel therapeutic agents based on these mechanisms.

Pyroptosis is a proinflammatory mode of programmed cell death characterized by cell swelling and eventual rupture and the release of inflammatory contents following the perforation of the cell membrane, which is mediated by the N-terminal domain of the gasdermin protein [5, 6]. The gasdermin family has six members, including GSDMA, GSDMB, GSDMC, GSDMD, GSDME (DFNA5), and DFNB59. Except for DFNB59, the pyroptosis-mediating ability of all members has been well validated [7]. The inflammatory response caused by pyroptosis promotes immune cell infiltration to eliminate the pathogen or activate the tumor microenvironment [8–10]. However, excessive inflammatory responses not only damage normal cells but also reduce immune surveillance and the suppression of malignant cells, resulting in chronic inflammation and tumor immune escape [11]. As an important molecular marker of pyroptosis [9], IL1*β* is generally released from the pore formed by oligomerized GSDMD [12] and has been shown to be closely associated with the formation of an immunosuppressive microenvironment in several tumor types [13–15]. Notably, several studies have shown that the proinflammatory cytokine IL1*β* is significantly elevated in the serum of glioblastoma (GBM) patients and serves as a potential serum marker for this type of disease [16, 17]. In addition, a very recent study showed that monocyte-derived macrophages in gliomas secrete IL1*β* in response to tumor cell induction, while *Il1b* knockdown significantly prolonged the survival time of primary glioma mice [18]. These studies suggest that aberrant pyroptosis signals may be present in glioma. In fact, pyroptosis has been demonstrated to be associated with the development of various peripheral inflammatory diseases and tumors, and several recent studies have reported that pyroptosis plays a critical role in the progression of CNS diseases, including Alzheimer's disease [19], multiple sclerosis [20], and stroke [21]. However, the relationship between pyroptosis and glioma, the most common primary tumor of the CNS, has rarely been reported. Here, we hypothesized that the progression of pyroptosis within gliomas could be used as a novel criterion for disease staging and prognostic assessment.

Although there have been several studies on prognosis-related pyroptosis genes in glioma [22, 23], they have been limited to establishing a prognostic model based on regression analysis while ignoring the correlation between pyroptosis and the tumor immune microenvironment. Moreover, these studies have tended to analyze all pyroptosis-related genes in general, but different gasdermin-mediated pyroptosis pathways are relatively independent from each other, and many genes are involved in other biological functions, such as apoptosis, so each pathway should be explored independently to reflect the situation of pyroptosis in glioma more accurately. The aim of this study was to identify the potential origin of pyroptosis activation signals in glioma through bioinformatics analysis of bulk RNA-seq data and single-cell RNA sequencing (scRNA-seq) data from glioma patients and to develop a risk score model based on markers of this signaling pathway to more effectively predict patient prognosis. This model will help to explore the relationship between pyroptosis and the progression of glioma. In addition, we explored the potential link between pyroptosis and the immune microenvironment in glioma. We also used single-cell transcriptomics data to identify cell clusters in tumors with the ability to induce pyroptosis, and these clusters can provide targets for the development of novel therapies for glioma.

## 2. Materials and Methods

### 2.1. Data Acquiring

The bulk RNA-seq and clinical information of glioma patients were obtained from TCGA database (https://portal.gdc.cancer.gov/), CGGA database (http://www.cgga.org.cn/), and GEO database (https://www.ncbi.nlm.nih.gov/geo/). TCGA cohort contained 702 tumor samples, the CGGA cohort contained 325 tumor samples, the Bao dataset (GSE48865) [24] contained 274 tumor samples, and the Gravendeel dataset (GSE12907, GSE4271) [25] contained 276 tumor samples. TCGA and CGGA cohorts are used for candidate gene screening and prognostic model establishment and validation, while the other cohorts are used for candidate gene screening only. TCGA and CGGA datasets used for prognostic modeling screened samples according to the following criteria: (1) having WHO grade classification and ≥II; (2) having complete survival information, including overall survival and final events; and (3) having not received immune checkpoint blocker therapy. There were 597 samples in the filtered TCGA dataset and 306 samples in the CGGA dataset, and detailed clinical characteristics are summarized in Table 1.

**Figure 1:**
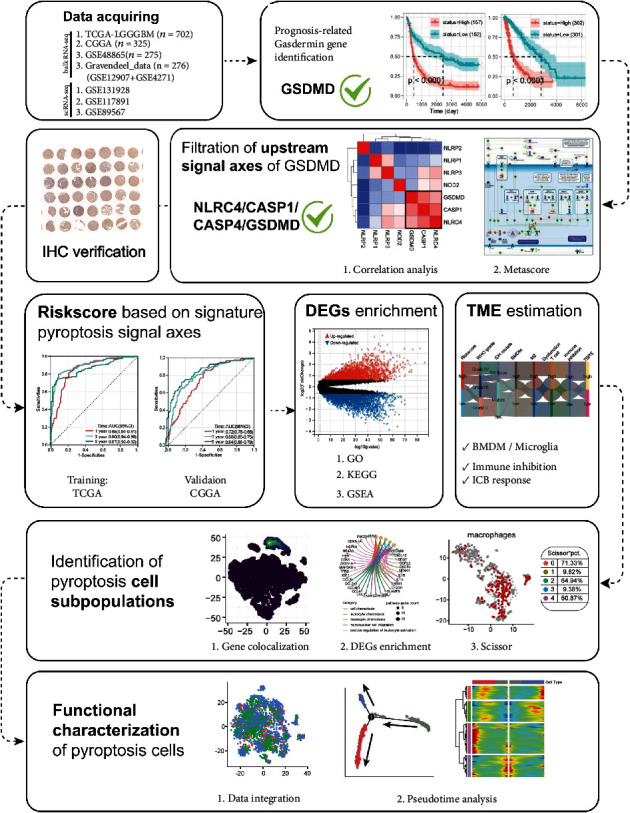
The complete research workflow. IHC: immunohistochemistry; DEGs: differentially expressed genes; GO: Gene Ontology analysis; KEGG: Kyoto Encyclopedia of Genes and Genomes; GSEA: gene set enrichment analysis; TME: tumor microenvironment; BMDM: bone marrow-derived macrophages; ICB: immune checkpoint blockade.

The scRNA-seq expression profiles and cell annotation files of Cyril Neftel et al. (GSE131928) [26], which contained a total of 7930 cells from 28 patients, were obtained from the Single Cell Portal database (https://singlecell.broadinstitute.org/single_cell). The scRNA-seq expression profiles of Kai Yu et al. (GSE117891) [27], which contained 6148 cells from 13 patients, were obtained from the CGGA database. The scRNA-seq expression profiles of Andrew Venteicher et al. (GSE89567) [28], which contained 6341 cells from 10 patients, were obtained from the GEO database. A simplified workflow for the current study is depicted in Figure (1).

### 2.2. Tumor Microenvironment Estimation

Immune score, stromal score, and ESTIMATE score were calculated using the ESTIMATE R package [29]. CIBERSORT was used to predict the abundance of each type of cell infiltration in the tumor microenvironment (TME) [30]. Dysfunctional CD8+ T cell infiltration levels for TCGA_LGG and TCGA_GBM were obtained from the Tumor Immune Dysfunction and Exclusion (TIDE) portal (http://tide.dfci.harvard.edu/) [31]. Response to immune checkpoint blockade therapy in TCGA and CGGA cohorts was also predicted in the TIDE portal. In addition, we used single sample gene set enrichment analysis (ssGSEA) to predict immune inhibition scores and TGF-*β* response scores (TBRS) in each sample based on the gene sets identified by Mariathasan et al. [32] (Supplementary Table. S1).

### 2.3. Differentially Expressed Gene Analysis and Functional Annotation

Analysis and functional annotation of differentially expressed genes (DEGs) were performed using an empirical Bayesian approach by using the limma R package. Adjusted *p* values less than 0.05 and absolute Log2 fold changes (log2FC) greater than 1.5 were considered DEGs and used for GO and KEGG functional annotation by the clusterProfiler R package.

### 2.4. Visualization of the Pyroptosis Pathway

To explore the GSDMD upstream signaling pathway, we divided TCGA cohort into the high- and low-expression groups by median GSDMD expression values and screened DEGs. The network building tool MetaCore™ version 5.4 (GeneGo) was used for pathway enrichment of differentially expressed genes as described previously [33], and the upstream signaling pathways of GSDMD were selected for visualization and analysis.

### 2.5. Tissue Microarray and Immunohistochemistry (IHC)

Tissue microarrays purchased from Bioaitech were used for IHC. Each microarray contained 11 normal brain tissues, 7 grade I glioma samples, 32 grade II glioma samples, 22 grade III glioma samples, and 36 grade IV glioma samples. Anti-human GSDMD-N antibody (abcam, ab215203), anti-human NLRC4 antibody (abclonal, A13117), and anti-human PD1 antibody (servicebio, GB12338) were used for staining. The degree of IHC staining was reviewed and scored independently by two observers who were unaware of the clinical characteristics. The intensity of staining was scored according to the following criteria: cells with <25% staining were scored as (−, 1); cells with 25-49% staining were scored as (+, 2); cells with 50-74% staining were scored as (++, 3); and cells with 75-100% staining were scored as (+++, 4). The staining color was scored as negative light-yellow particle (1), brown-yellow particle (2), and brown particle (3). The final score was defined as the staining number score multiplied by the staining color score.

### 2.6. Generation of Riskscore

To establish a risk score that can assess the degree of activation of apoptotic pathways in individual patients, we performed multivariate Cox analysis on the screened highly conserved co-expressed gene cluster NLRC4/CASP1/CASP4/GSDMD using TCGA cohort as a training set. The Riskscore formula was constructed based on the coefficients of multivariate Cox analysis and validated for stability in the CGGA cohort. Kaplan–Meier curves were plotted to prove the prognostic value of the Riskscore, and log-rank tests were employed for analyzing statistical differences between the high- and low-risk groups. The accuracy of the Riskscore was assessed using receiver operating characteristic (ROC) curves. The independence of Riskscore was assessed using univariate and multifactorial Cox analyses.

### 2.7. Copy Number Variation and Tumor Mutational Burden Analysis

To determine copy number alteration events, we used the set of discrete copy number calls provided by GISTIC 2.0: homozygous deletion (−2); hemizygous deletion (−1); no-change (0); low-level gain (1); and high-level amplification (2). When more than half of the genes in the amplified or deleted peak region were high-level amplification (2) or homozygous deletion (−2), the copy number of the peak region is defined as changed. The oncoplot function in the maftools R package was used to visualize the general condition of the Mutation Annotation Format (MAF) of TCGA cohort in the form of a waterfall chart.

### 2.8. Bone Marrow-Derived Macrophage (BMDM) and Microglia Estimation

We used the single sample gene set enrichment analysis (ssGSEA) to predict BMDM and microglia infiltration scores in each sample based on the DEGs between microglia and BMDM demonstrated by Bowman et al. [34] as gene sets (Supplementary Table. S2). The gene set of DEGs in BMDM and microglia identified by Muller et al. [35] was used to validate the robustness of the above prediction. The same approach was used to predict the BMDM and microglia infiltration scores of macrophage subpopulations in the scRNA-seq dataset to distinguish BMDM and microglia at the single-cell level.

### 2.9. scRNA-seq Data Processing

The Seurat R package was used for scRNA-seq data processing as previously described [36]. Cells were removed if the number of expressed genes was less than 200 or more than 6,000, the UMI count was less than 1,000 and/or the percentage of mitochondrial genes was more than 0.1. The NormalizeData and ScaleData functions are used to normalize the matrix for subsequent cell clustering and dimensionality reduction. The first 2,000 highly variable genes identified by the FindVariableFeatures function were used in the RunPCA function for principal component analysis (PCA). The FindClusters function is used to cluster cells at a resolution of 0.5. RunTSNE is used to project cells into two dimensions and visualize them. The FindAllMarkers function was used to identify specific macrophage cluster DEGs compared to all other macrophage clusters. The harmony R package was used for integration and batch effect correction of expression profiles of BMDM and microglia from different datasets [37]. The monocle R package was used for performing differential expression and time-series analysis for single-cell expression experiments.

### 2.10. Identifying Phenotype-Associated Subpopulations

As previously described, the Scissor R package was used for phenotype-guided single-cell subpopulation identification [38]. Briefly, the Cyril Neftel single-cell expression matrix, TCGA bulk expression matrix, and phenotype of interest (overall survival in this study) were processed using Scissor. All cells can be divided into Scissor-positive (Scissor^+^) cells and Scissor-negative (Scissor^−^) cells, which are positively and negatively associated with the phenotype of interest, respectively.

### 2.11. Statistical Analysis

All statistical analyses were performed using R (4.1.2) software. Student's *t*-test (unpaired, two-tailed) was used to assess differences between two independent groups, and the Wilcoxon test is used for nonparametric tests between data that do not conform to a normal distribution. One-way analysis of variance (ANOVA) was used as a parametric method for data from more than two groups. The chi-square test was executed for the comparison of categorical variables between the high- and low-risk groups. The survivor and survminer R packages were used for survival analysis.

## 3. Results and Discussion

### 3.1. GSDMD Significantly Correlated with the Progression and Overall Survival of Glioma

Gasdermin proteins are the final executors of pyroptosis, and their expression level directly affects the possibility of pyroptosis occurring [39]. Considering that the gasdermin family contains five members that have been confirmed to mediate pyroptosis, we examined the relationship between the different gasdermin genes and the progression or prognosis of glioma to investigate the most critical gene for the execution of pyroptosis in gliomas.

**Figure 2:**
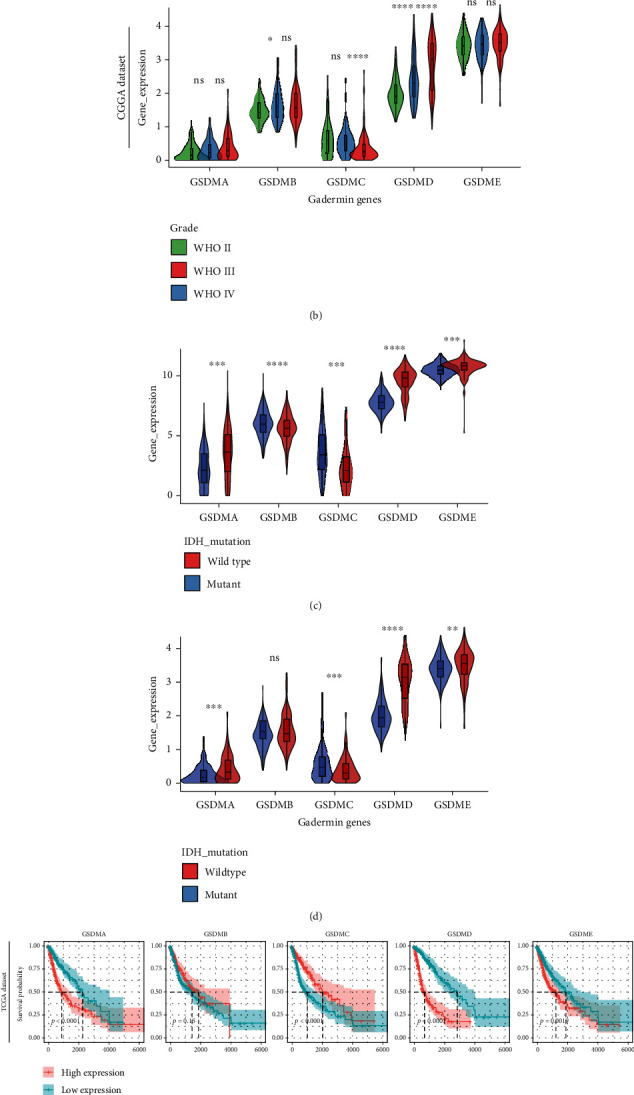
GSDMD expression was significantly associated with the progression and prognosis of glioma. (a, b) Expression levels of gasdermin family genes in patients with different WHO grades in TCGA and CGGA cohorts. (c, d) Expression levels of gasdermin family genes in patients with different IDH mutation phenotypes in TCGA and CGGA cohorts. (e, f) Kaplan–Meier plots for overall survival time (OS) of patients with different gasdermin family gene expression in TCGA and CGGA cohorts, using the median expression of each gene as a cutoff point to divide the high and low expression groups. Statistics were calculated using two-tailed, unpaired Student's *t*-test with Welch's correction in a-d. ns: not significant. ^∗^*p* < 0.05,  ^∗∗^*p* < 0.01,  ^∗∗∗^*p* < 0.001, and^∗∗∗∗^*p* < 0.0001.

We first analyzed the expression of gasdermin family genes in different-grade gliomas in TCGA-LGGGBM and CGGA cohorts. The results showed that among the five pyroptosis genes of this family, only the expression of GSDMD showed a stable correlation with disease progression in both cohorts, and higher WHO grades corresponded to higher GSDMD expression (Figures 2(a) and 2(b)). This finding is consistent with the findings reported by Liu et al. that (1) GSDMD protein levels were elevated in clinical glioma tissue, accompanied by significant cleavage bands, and (2) GSDMD protein expression in GBM samples was higher than that in LGG samples [40]. IDH represents a major biomarker with diagnostic, prognostic, and predictive implications in glioma, and mutant phenotypes have a worse prognosis (Figure S1). The expression of multiple gasdermin genes in TCGA and CGGA cohorts was significantly different among the IDH phenotype groups (Figures 2(c) and 2(d)). However, in TCGA cohort, the expression of GSDMD (wild type vs. mutant) Log2FC = 1.784 was compared with the expression of gasdermin Log2FC in -0.823~0.899; in the CGGA cohort, the expression of GSDMD (wild type vs. mutant) Log2FC = 1.603 was compared with the expression of gasdermin Log2FC in -0.553~0.815. Therefore, among the gasdermin family members, GSDMD is the most differentially expressed gene among different IDH phenotypes of glioma.

To further confirm that GSDMD has a more significant indicative role in glioma than other gasdermin genes, we evaluated the overall survival time of each group based on the clinical information of patients from TCGA and CGGA datasets and gasdermin gene expression profiles, using the median expression of each gene as a cutoff point to divide the high and low expression groups (Figures 2(e) and 2(f)). The results showed that GSDMA, GSDMC, and GSDMD were significantly correlated with prognosis in both TCGA and CGGA datasets and high expression of GSDMA and GSDMD corresponded to shorter survival, but GSDMC showed the opposite results. However, GSDMB and GSDME showed only limited prognostic relevance in a single dataset. Notably, in TCGA and CGGA cohorts, compared to the high expression group, the median survival time was prolonged 4.36-fold and 5.22-fold in the GSDMD low-expression group, while it was prolonged only 2.38-fold and 1.63-fold in the GSDMA low-expression group.

In conclusion, multiple gasdermin-mediated complex pyroptosis signaling networks may exist in gliomas. However, compared with other gasdermin genes, the pyroptosis triggered by GSDMD plays the most critical role in both the progression and the prognosis of glioma. Therefore, we performed subsequent data mining work around GSDMD.

### 3.2. The NLRC4/CASP1/CASP4/GSDMD Pyroptosis Signaling Axis Can Be Used as a Prognostic Factor for Glioma

**Figure 3:**
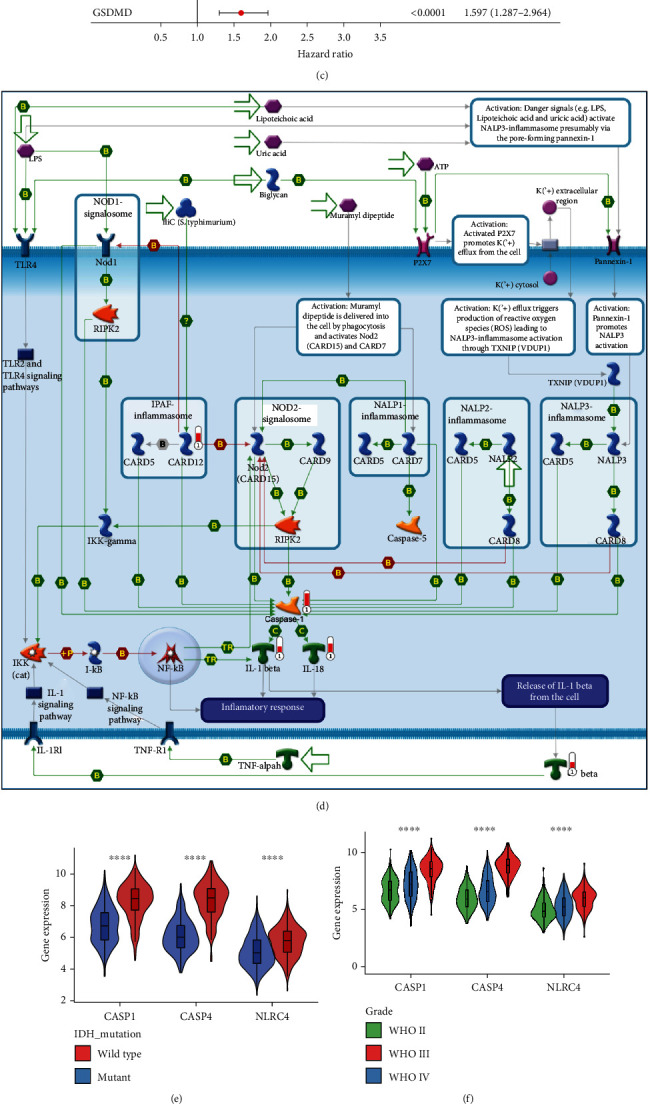
NLRC4/CASP1/CASP4/GSDMD signaling axis genes in TCGA cohort have similar expression patterns and jointly affect overall survival. (a) Kaplan–Meier plots for the OS of patients with different CASP gene expression in TCGA cohort, using the median expression of each gene as a cutoff point to divide the high- and low-expression groups. (b) Scatter plot of the correlation between the GSDMD gene and the expression of three caspase genes in TCGA cohort. The degree of correlation was examined using Spearman's coefficient. (c) Univariate Cox analysis of the effects of GSDMD, CASP4, and CASP1 and their upstream inflammasome gene expression on overall survival. Genes with *p* < 0.05 were selected for multivariate Cox analysis. Hazard ratios are presented as forest plots. (d) Pathway enrichment of DEGs between the GSDMD high- and low-expression groups using Metacore and visualization of CASP1 upstream gene hits. The red thermometer indicates the Log2FC of different genes. (e, f) Expression levels of CASP1, CASP4, and NLRC4 genes in patients with different WHO grades and IDH mutation phenotypes in TCGA cohort. (g) Representative sections of matched GSDMD-N and NLRC4 immunohistochemistry from normal brain tissue and different grades of glioma samples. (h, i) Semiquantitative results of GSDMD-N and NLRC4 staining levels in tissue microarrays. Statistics were calculated using one-way analysis of variance (ANOVA) in (f). Statistics were calculated using two-tailed, unpaired Student's *t*-test with Welch's correction in (e, h, i). ^∗∗∗∗^*p* < 0.0001,  ^∗∗^*p* < 0.01, and^∗^*p* < 0.05.

The process of pyroptosis requires not only gasdermin expression but also upstream activation signals leading to gasdermin cleavage and N-terminal domain release, which are equally crucial. It has been well demonstrated that caspase-1, caspase-4, and caspase-5 are activators of GSDMD, capable of cleaving GSDMD at hGSDMD_276_ and releasing GSDMD-NT, leading to pyroptosis [5]. CASP1, CASP4, and CASP5 were all significantly associated with overall survival in TCGA cohort (Figure 3(a)). Among them, CASP1 and CASP4 showed a particularly significant positive correlation with GSDMD (*R* > 0.75, *p* < 10^−16^), while the coexpression of CASP5 was discrete (Figure 3(b)). The highly conserved coexpression relationships between CASP1/GSDMD and CASP4/GSDMD were validated in three other independent datasets (Figure S2), indicating CASP1/CASP4/GSDMD signaling axis activation in glioma.

Inflammasomes are the activator of multiple caspases, among which multiple inflammasomes such as NLRP1, NLRP3, and NLRC4 are directly involved in classical or nonclassical pyroptosis pathways and act as receptors of pyroptosis [41]. Therefore, to explore the upstream activation signals of CASP genes, we selected representative NLRP1, NLRP2, NLRP3, NLRC4, and NOD2 inflammasome genes to examine their correlation with CASP1/CASP4/GSDMD. Univariate Cox regression analysis revealed that only NLRC4 of the CASP1 upstream inflammasome genes had a significant effect on overall survival (HR = 2.594, *p* < 0.0001), and multivariate Cox regression analysis demonstrated that NLRC4/CASP1/CASP4/GSDMD could jointly affect overall survival in TCGA cohort (Figure 3(c)). Among inflammasome genes, NLRC4 demonstrated its unique prognostic value in the CGGA cohort and Gravendeel dataset (Figure S3). Furthermore, we screened differentially expressed genes (DEGs) between the high and low GSDMD expression groups using the limma R package, performed pathway enrichment of DEGs, and analyzed GSDMD upstream gene hits using the network building tool MetaCore. The visualization results again demonstrated the unique association of the NLRC4 (CARD12 in the map) gene with GSDMD among inflammasome genes (Figure 3(d)). In addition, the expression of the CASP1, CASP4, and NLRC4 genes in TCGA cohort increased with disease progression and showed higher expression in the wild-type IDH group (Figures 3(e) and 3(f)), and the NLRC4/CASP1/CASP4/GSDMD coexpression relationship was verified in TCGA cohort and three other independent datasets (Figure S4). To further confirm pyroptosis in gliomas, we examined the protein levels of N-terminal GSDMD (GSDMD-N), which is produced by cleavage of full-length GSDMD by caspase-1 and caspase-4 and is the most classic marker of pyroptosis, in gliomas of different disease grades and normal brain tissue. We also examined the protein levels of NLRC4. The results showed that GSDMD-N and NLRC4 were barely detectable in the normal brain tissue, while the levels of both GSDMD-N and NLRC4 increased in matched samples with increasing disease grade (Figure 3(g)). Semiquantitative analysis also showed significantly higher levels of GSDMD-N and NLRC4 in higher-grade glioma samples (Figures 3(h) and 3(i)), which provided conclusive evidence for GSDMD-mediated pyroptosis in gliomas.

**Figure 4:**
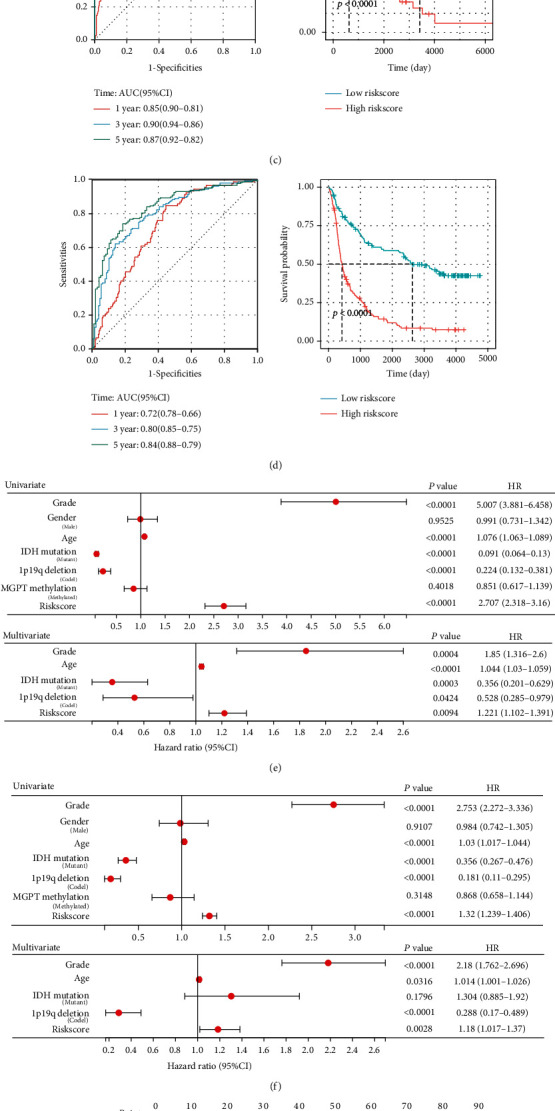
Stable prognostic model based on NLRC4/CASP1/CASP4/GSDMD pyroptosis signaling axis genes. (a, b) Relationship between risk score and overall survival of patients and expression levels of NLRC4, CASP1, CASP4, and GSDMD genes in TCGA training cohort and CGGA validation cohort. (c, d) Time-dependent ROC analysis and Kaplan–Meier analysis in TCGA training cohort and CGGA validation cohort to assess the prognostic value of the Riskscore, using the median Riskscore as a cutoff point to divide the high- and low-risk groups. (e, f) Univariate Cox analysis and multivariate Cox analysis in TCGA training cohort and CGGA validation cohort. Hazard ratios are presented as forest plots. (g, h) The nomogram for predicting the proportion of patients with 1-, 3-, and 5-year overall survival in TCGA and CGGA cohorts. (i, j) The calibration curves for the prediction of 1-, 3-, and 5-year overall survival in TCGA cohort and CGGA cohort.

To investigate in depth whether this signaling axis can be used as a valid guide for predicting patient prognosis, we constructed a scoring system based on the NLRC4/CASP1/CASP/GSDMD pyroptosis axis. We extracted the 4 genes with significant coefficients in the multivariate Cox analysis of TCGA dataset, used these data as the training set, and finally obtained the risk score formula: Riskscore = 1.02065 × expr_CASP4_ + 0.46802 × expr_GSDMD_ + 0.31184 × expr_CASP1_ + 0.09562 × expr_NLRC4_. The Riskscore for each patient was calculated, and the patients were divided into the high- and low-risk groups according to the median Riskscore (Figures 4(a) and 4(b)). There were significant differences in the histological classification, WHO grade, IDH mutation phenotype, and chromosome 1p19q codeletion phenotype between the two groups, while chemotherapy and temozolomide acceptance differences were not significant (Table.  1). Time-dependent ROC and Kaplan–Meier curves were used to assess the prognostic ability of the four pyroptosis-associated genetic signatures. The results showed that the high-pyroptosis-risk group defined by the four signature genes had significantly shorter OS in TCGA training cohort and the CGGA external validation cohort (Figures 4(c) and 4(d)). The AUC (area under the ROC curve) was 0.85, 0.90, and 0.87 for the 1-year, 3-year, and 5-year OS in the training cohort and 0.72, 0.80, and 0.84 in the CGGA cohort, respectively. The ROC curves showed a similar prognostic value of our established prognostic model and the previously established 10-pyroptosis-gene prognostic model [22] and golden standard WHO grading system for predicting OS at 1, 3, and 5 years in TCGA and CGGA cohorts (Figure S5). Moreover, univariate and multifactorial Cox regression analyses revealed that the Riskscore could be used as a valid independent prognostic factor, as well as disease grade, age, IDH mutation status, and 1p19q codeletion status (Figures 4(e) and 4(f)). Nomograms based on the results of multivariate Cox regression analysis were used for scoring to assess the accuracy of the model. To correctly predict the 1-, 3-, and 5-year OS, we created a nomogram4 that included the WHO grade, age, IDH mutation status, 1p19q codeletion status, and the Riskscore (Figures 4(g) and 4(h)). The calibration curve study revealed agreement between the patients' anticipated and observed 1-, 3-, and 5-year OS rates in both TCGA and CGGA cohorts (Figures 4(i) and 4(j)).

### 3.3. Differential Gene, Tumor Mutational Burden, and Drug Prediction Analysis Based on the Four-Pyroptosis-Gene Prognostic Model

**Figure 5:**
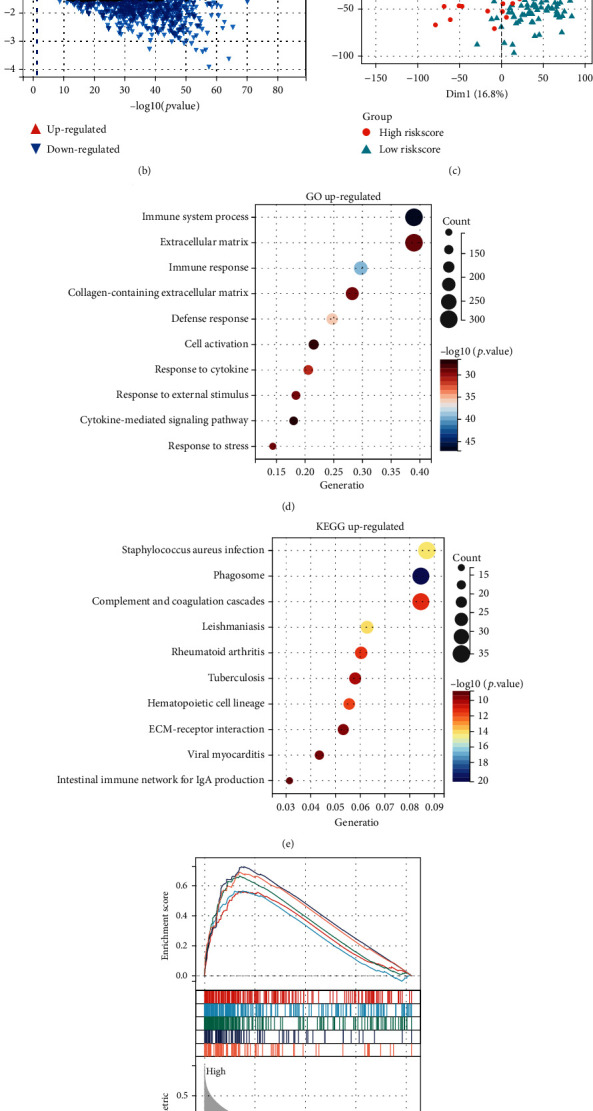
Upregulated DEGs were associated with inflammatory responses, and downregulated DEGs were associated with synaptic stabilization. (a) Heatmap of DEG expression profiles in TCGA cohort with clinical phenotypes, including the disease grade, IDH phenotype, Riskscore, and risk group. (b) Volcano map of DEGs between the two risk groups in TCGA cohort. (c) Principal component analysis (PCA) of all patients in TCGA cohort based on all gene expression profiles between risk groups. (d, e) Bubble plots of GO and KEGG enrichment analysis for upregulated DEGs. (f) HALLMARK terms were used for GSEA enrichment analysis based on the Riskscore of all patients in TCGA cohort, with the enriched terms represented by different color curves. (g) Bubble plot of GO enrichment analysis for downregulated DEGs.

The distinct prognosis of the high- and low-risk groups defined by the four pyroptosis genes drove us to further explore the functional enrichment of the differential genes between the high- and low-risk groups and thus speculate on the potential mechanisms of pyroptosis involved in glioma disease progression. We analyzed differentially expressed genes between the high- and low-risk groups in TCGA dataset using the Limma R package. We screened DEGs with ∣log2‐fold change | >1.5 and adjusted *p* < 0.05 and obtained a total of 498 upregulated genes and 518 downregulated genes (Figures 5(a) and 5(b)). Principal component analysis (PCA) showed that the high-risk group distinctly clustered apart from the low-risk group, revealing significant differences in expression profiles between the two groups (Figure 5(c)).

The GO enrichment results showed that upregulated DEGs were mainly involved in various immune response-related biological processes, such as cellular immune response, cellular defense response, and response to cytokines (Figure 5(d)). The KEGG enrichment results indicated that upregulated DEGs were mainly associated with inflammatory response signals, such as multiple bacterial and viral infections and autoimmune diseases such as rheumatoid arthritis (Figure 5(e)). Gene set enrichment analysis (GSEA) also showed the activation of various proinflammatory signaling pathways in the high-risk group, including IL2-STAT5, IL6-STAT3, and IFN-*α* responses (Figure 5(f)). Pyroptosis is a proinflammatory cell death mode in which large amounts of inflammatory substances are released during cell death and trigger an inflammatory response, which coincided with the activation of multiple aberrant immune response pathways present in the high-pyroptosis-risk group. In addition, the top ten GO terms enriched by downregulated DEGs all had a strong link with synapse formation, stabilization, and signal transduction (Figure 5(g)). Clinically, patients with high-grade glioma tend to develop degenerative diseases such as memory loss and cognitive impairment, and it has been demonstrated that impaired cognitive function is associated with shorter survival in glioblastoma patients [42]. Furthermore, pyroptosis is strongly associated with Alzheimer's disease progression, and GSDMD serves as an important marker for AD [19]. This is consistent with our analysis that there is an association between high-grade gliomas, corresponding to a high risk of pyroptosis, and neurodegenerative diseases. Taken together, these results indicate that a high degree of CASP1/CASP4/NLRC4/GSDMD pyroptosis is accompanied by the activation of proinflammatory signaling pathways in the brain and is closely associated with impaired establishment and stability of neuronal synapses.

We also explored differences in copy number variation (CNV) and tumor mutational burden (TMB) between the high-risk and low-risk groups. A significantly higher proportion of samples in the high-risk group had CNV. We screened for the genes that differed most significantly between the high-risk and low-risk groups, including high-level amplified genes and homozygous deletion genes. Interestingly, several interferon alpha (IFNA) family genes in the high-risk group were homozygously deleted (Figure S6a), and the activation of the inflammasome was previously reported to have an antagonistic effect on the type I interferon response in macrophages [43]. However, a correlation of other genes with pyroptosis could not be identified in previous studies. In addition, the CNV in the NLRC4, CASP1, CASP4, and GSDMD genes did not vary significantly between the high- and low-risk groups (Figure S6b). The low-risk group had an IDH1 mutation rate of 86.95%, and the majority of these samples also had mutations in the ATRX and CIC genes, which are characteristic of LGGs, such as oligodendrogliomas [44] (Figure S6c). In contrast, more EGFR, TTN, and PTEN mutations, which are usually characteristic of GBM [45], were observed in the high-risk group (Figure S6d).

We also performed a preliminary drug sensitivity analysis. The drug sensitivity data and expression profile data for glioma cell lines were obtained from Genomics of Drug Sensitivity in Cancer (GDSC) and the Cancer Therapeutics Response Portal (CTRP). Multiple drug candidates were screened by correlation analysis of the expression levels of the four pyroptosis genes of the cell lines with the IC50 of different drug treatments in the GDSC database (Figure S7a, b) and the drug sensitivity (1-(AUC/30)) in the CTRP database (Figure S7c, d). However, the sensitivity to each of these drug candidates can only be correlated with the expression of one of the pyroptosis genes, so the combination is more appropriate for this pyroptosis target.

### 3.4. Increased Infiltration of BMDMs and the Immunosuppressive Microenvironment in the High-Risk Group

**Figure 6:**
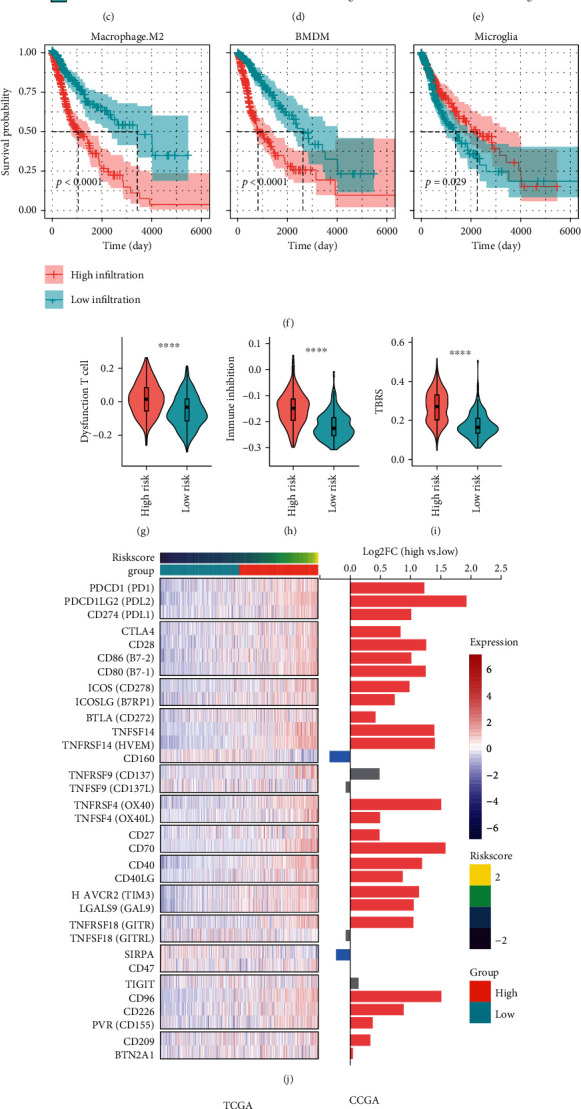
The Riskscore, defined by the pyroptosis axis, was associated with the immunosuppressive tumor microenvironment. (a) Stromal score, immune score, and ESTIMATE score of TCGA cohort predicted based on ESTIMATE R package. (b) Immune cell infiltration in TCGA cohort predicted by CIBERSORT. (c) ssGSEA based on macrophage and microglial DEGs to calculate the BMDM infiltration score and microglial infiltration score for each sample. (d) Scatter plots of the correlation between the infiltration score of BMDMs or microglia and the infiltration of M2-type macrophages. Spearman's coefficient was used to evaluate the degree of correlation. (e) Scatter plots of the correlation between the infiltration fraction of BMDMs or microglia and the Riskscore. Spearman's coefficient was used to evaluate the degree of correlation. (f) Kaplan–Meier curves for the correlation between M2 macrophage, BMDM, and microglial infiltration and overall survival time in TCGA cohort, using the median infiltration score as a cutoff point to divide the high- and low-infiltration groups. (g) Dysfunctional CD8+ T cell infiltration score predicted by Tumor Immune Dysfunction and Exclusion (TIDE) in TCGA cohort. (h, i) Immunosuppression scores (h) and TGF-*β* response score (TBRS) (i) calculated for each sample based on ssGSEA with different characteristic gene sets. (j) Heatmap of the expression of immune checkpoint genes in TCGA cohort, aligned by immune checkpoint gene pairing and displaying the Log2FC (high-risk vs. low-risk) of the corresponding gene expression on the *y*-axis. The results for *p* > 0.05 are shown in gray. (k) The ICB responses of TCGA (left) and CGGA cohorts (right) based on the TIDE prediction results are presented in the stacked histograms. (l) Representative sections of matched GSDMD-N and PD1 immunohistochemistry from high pyroptosis group (GSDMD-N score > 5) and low pyroptosis group (GSDMD-N score < 5). (m) Semiquantitative results of PD1 staining levels in tissue microarray. Statistics were calculated using two-tailed, unpaired Student's *t*-test with Welch's correction in (a–c) and (g–i). Statistics were calculated using the Chi-squared test in (k). ns: not significant. ^∗^*p* < 0.05,  ^∗∗^*p* < 0.01,  ^∗∗∗^*p* < 0.001, and^∗∗∗∗^*p* < 0.0001.

We demonstrated that the high-risk group was associated with multiple inflammatory response signaling pathways (Figures 5(e)–5(g)), and we speculated that this may be associated with the altered infiltration of immune cells caused by the NLRC4/CASP1/CASP4/GSDMD pyroptosis axis. The ESTIMATE R package was used to predict the stromal score, immune score, and ESTIMATE score (stromal score + immune score), and the results showed that the high-risk group had a higher immune score and stromal score, which represented a higher degree of immune infiltration and tumor malignancy (Figure 6(a)). CIBERSORT was used to predict immune cell infiltration in TCGA cohort, and the most abundant immune cells in gliomas were M2 macrophages, which were further increased in the high-risk group. (Figure 6(b)). Previous studies have shown that macrophages in gliomas, especially those with the M2 phenotype, play an important role in the formation of the immunosuppressive microenvironment and tumor progression [46, 47]. Since brain macrophages can be divided into bone marrow-derived macrophages (BMDMs) and tissue-resident microglia and function differently, we used the DEGs between microglia and BMDMs demonstrated by Bowman et al. (Supplementary Table. S2) [34] as gene sets and assessed the microglia and BMDM infiltration in each sample by ssGSEA (single sample GSEA). BMDM infiltration differed remarkably between the high- and low-risk groups, while microglia did not change significantly (Figure 6(c)). Notably, the BMDM infiltration score had a significant positive correlation with M2 macrophage infiltration (*R* = 0.56, *p* < 0.0001) (Figure 6(d)) and the Riskscore (*R* = 0.69, *p* < 0.0001) (Figure 6(e)), which could not be observed in microglia. In addition, high infiltrations of M2 macrophages and BMDMs were strongly associated with poor prognosis, whereas microglia were of opposite and limited predictive value (*p* = 0.029) (Figure 6(f)). To verify the robustness of the association of high BMDM infiltration with poor prognosis and the positive correlation between BMDM infiltration and the Riskscore, we further used the microglia and BMDM differentially expressed genes from the study of Muller et al. (Supplementary Table. S3) to predict microglial and BMDM infiltration scores [35]. The results once again demonstrated the excellent prognostic value of BMDM infiltration, but not microglial infiltration (Figure S8b), and the robust positive correlation between BMDM infiltration and the Riskscore (*R* = 0.75, *p* < 0.0001) or M2 macrophage infiltration (*R* = 0.62, *p* < 0.0001) (Figure S8c, d).

Dysfunctional CD8+ T cell infiltration predicted by Tumor Immune Dysfunction and Exclusion (TIDE) was higher in the high-risk group (Figure 6(g)). ssGSEA based on the immune checkpoint gene set and the TGF-*β* response score (TBRS) gene set associated with the anti-PD1 treatment response identified by Sanjeev et al. (Supplementary Table. S1) [32] was used to evaluate the immunosuppression score (Figure 6(h)) and TBRS (Figure 6(i)) in each sample, which were also higher in the high-risk group, representing a hypersuppressed immune microenvironment and disappointing anti-PD1 treatment response rate. Due to the lack of open access to immune checkpoint blockade (ICB) glioma therapy cohorts, we used TIDE to predict the response to ICB therapy in TCGA and CGGA cohorts. Response rates were significantly lower in the high-risk group than in the low-risk group in both cohorts (Figure 6(k)), consistent with previous results of a higher TBRS in the high-risk group. In addition, the landscape analysis of immune checkpoint receptor and ligand genes demonstrated a positive correlation between gene expression and the Riskscore for the majority of immune checkpoints (Figure 6(j)).

In addition, we divided the samples into the positive pyroptosis group (GSDMD-N score > 5) and the negative pyroptosis group (GSDMD-N score < 5) based on the staining results of GSDMD-N of tissue microarray and compared the PD1 immunohistochemical staining levels in the two groups. The results showed that the PD1 level in the positive pyroptosis group was significantly higher than that in the negative pyroptosis group (Figures 6(l) and 6(m)), indicating that pyroptosis in the microenvironment of glioma was accompanied by deepening immunosuppression. Therefore, it is hypothesized that inhibition of pyroptosis in glioma may facilitate the alleviation of the immunosuppressive microenvironment.

Although the Riskscore was previously demonstrated to be associated with proinflammatory signals (Figure 5), it is not contradictory to mediating the establishment of a suppressive immune microenvironment. Pyroptosis is a type of proinflammatory cell death, and prolonged infiltration of IL1*β*, IL2, and IL6 in the inflammatory environment induces Tregs [48] and promotes the invasion and proliferation of glioma stem cells (GSCs) [49]. Our study showed that the activation of the NLRC4/CASP1/CASP4/GSDMD pyroptosis axis was significantly and positively correlated with M2-type BMDM infiltration, suggesting that blocking pyroptosis in glioma may be a potential approach to reduce macrophage infiltration. Pyroptosis inhibitors have potential as adjuvant therapeutic agents in high-grade glioma, such as dimethyl fumarate, which has been approved by the FDA to reduce macrophage infiltration by inhibiting pyroptosis to achieve efficacy in the treatment of multiple sclerosis [20] and has been demonstrated to cross the blood–brain barrier [50].

### 3.5. The Activation of the NLRC4/CASP1/CASP4/GSDMD Signaling Axis Is Mainly Present in Peripheral BMDMs

**Figure 7:**
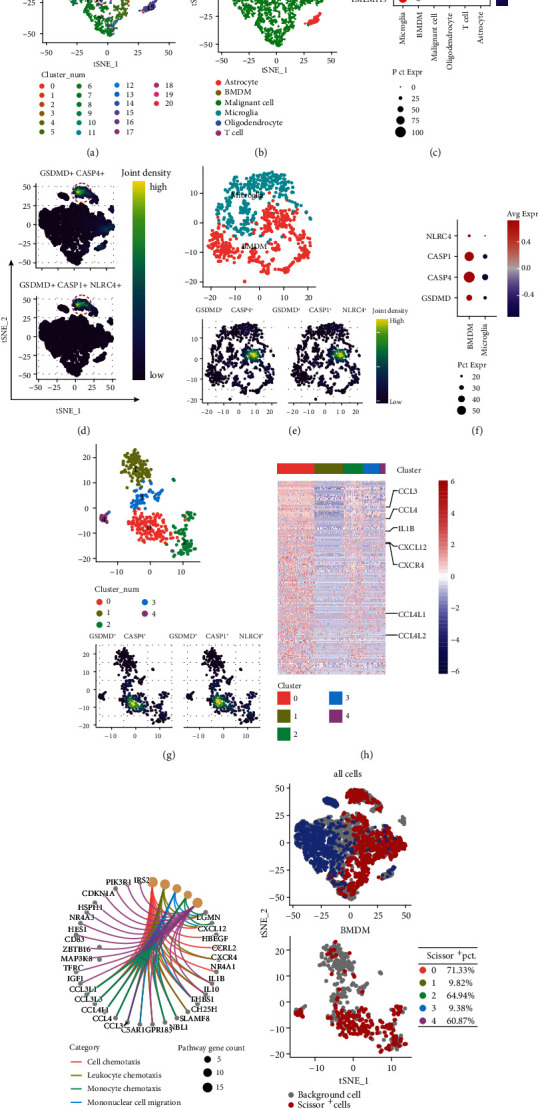
Single-cell transcriptomics revealed that the NLRC4/CASP1/CASP4/GSDMD pyroptosis axis was colocalized in peripheral-derived BMDMs. (a) *t*-distributed stochastic neighbor embedding (tSNE) plot of all single cells. (b) tSNE plot of all cells with cell-type annotations. (c) The signature gene expression matrix for cell cluster identification. (d) tSNE plot of CASP4+ GSDMD+ and NLRC4+ CASP1+ GSDMD+ cells in all cells. Red circles highlight the colocation cluster. (e) tSNE plots of microglia and peripheral-derived macrophages (BMDMs) (top) and distribution of CASP4+ GSDMD+ and NLRC4+ CASP1+ GSDMD+ cells in microglia and BMDMs (bottom). (f) Comparison of NLRC4, CASP1, CASP4, and GSDMD gene expression in BMDMs and microglia. (g) tSNE plots of the distribution of subpopulations of BMDMs (top) and distribution of CASP4^+^ GSDMD+ and NLRC4+ CASP1+ GSDMD+ cells in BMDM subpopulations (bottom). (h) Heatmap of DEG expression used to distinguish macrophage clusters_0~clusters_4 and highlight genes of interest on the right. (i) GO enrichment analysis of DEGs of BMDM cluster_0. (j) tSNE plots with scissors prediction results of all cells (top) and BMDM (bottom). The percentage of scissor-positive cells in each subpopulation of BMDMs is shown on the right.

To elucidate the potential link between macrophage infiltration and the NLRC4/CASP1/CASP4/GSDMD pyroptosis signaling axis, we further explored the source cells of pyroptosis in gliomas at single-cell resolution. The scRNA-seq expression profiles and cell annotation files of Neftel et al. [26] were obtained from the Single Cell Portal database, containing a total of 7930 cells from 28 patients (Figure 7(a)). After data cleaning and cell type clustering, cells were classified into malignant cells, macrophages, T cells, oligodendrocytes, and astrocytes (Figures 7(b) and 7(c)). ssGSEA predicted the BMDM score and microglia score in the same way as presented previously and was used to differentiate between peripheral-derived BMDMs and tissue-resident microglia (Figure S9).

The coexpression of CASP and GSDMD is required for the occurrence of pyroptosis, while CASP4, GSDMD and NLRC4, CASP1, and GSDMD colocalization signals were located in the macrophage/microglia population (Figure 7(d)) and were significantly concentrated in the BMDM cluster (Figure 7(e)). The gene expression levels of NLRC4, CASP1, CASP4, and GSDMD were all higher in BMDMs than in microglia (Figure 7(f)), suggesting that BMDMs are more sensitive to pyroptosis or more prone to pyroptosis than other cells. The BMDM cluster was further divided into five subpopulations from cluster_0 to cluster_4, while CASP4+ GSDMD+ and NLRC4+ CASP1+ GSDMD+ cells were clearly concentrated in the cluster_0 cell cluster (Figure 7(g)). With Log2FC > 1.5 and adjusted *p* < 0.05, 118 signature genes of the cluster_0 BMDM subpopulation were screened, including various chemokine genes, such as CCL3, CCL4, and CXCL12, and the proinflammatory cytokine gene IL1B (Figure 7(h)). There is no doubt that GO enrichment analysis hits BP terms of multiple cell chemotaxis and migration-related pathways (Figure 7(i)). Therefore, the positive cycle that accompanies the release of inflammatory molecules such as DAMPs and multiple chemokines during macrophage pyroptosis leads to more infiltration of peripheral monocytes and macrophages, which may trigger a worse prognosis. To use big data and survival information to aid in scRNA-seq data analysis, we used the Scissor R package to assess the relevance of single cells to patient overall survival. Briefly, using TCGA-LGGGBM dataset and the Cyril Neftel scRNA-seq dataset, Scissor was applied to distinguish cells with high expression of survival-related genes, where scissor-positive (Scissor^+^) cells were those associated with poor survival and scissor-negative (Scissor^−^) cells were those associated with good prognosis. As expected, the macrophage population was filled with a large number of cells associated with poor survival, and the NLRC4+ CASP1+ CASP4+ GSDMD+ BMDM concentrated cluster had a very high proportion of cells associated with a poor prognosis (71.33%) (Figure 7(j)).

**Figure 8:**
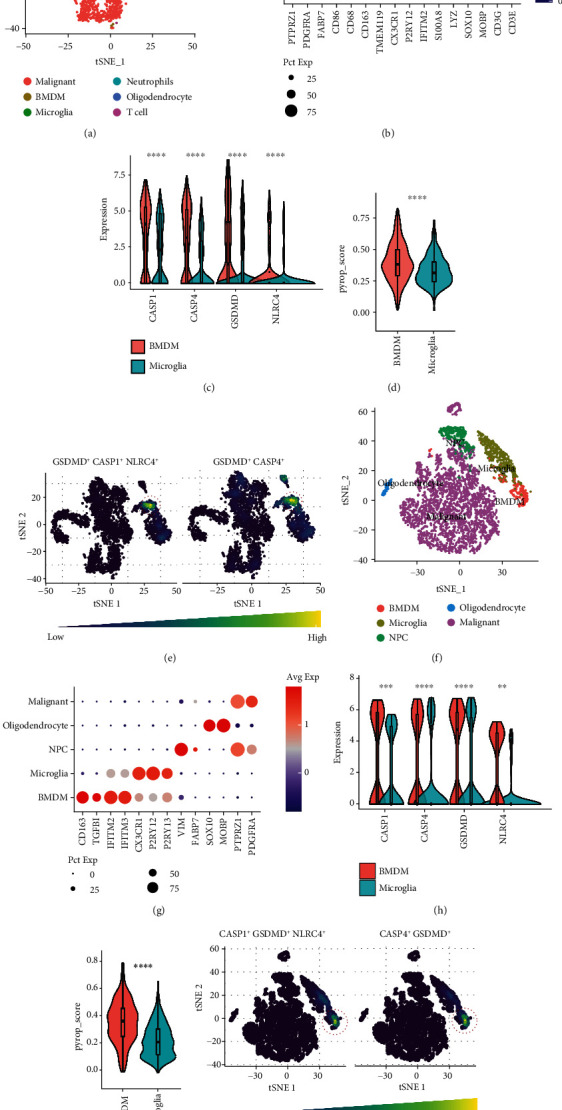
Analysis of other datasets confirmed the high expression of pyroptosis genes in BMDMs. (a–e) Visualization and analysis of the GSE117891 dataset. (a) *t*-distributed stochastic neighbor embedding (tSNE) plot of all single cells with cell type annotations. (b) The signature gene expression matrix for cell cluster identification. (c) Comparison of CASP1, CASP4, GSDMD, and NLRC4 expression in BMDMs and microglia. (d) Comparison of the PyropScore between BMDMs and microglia, which was calculated using ssGSEA based on the expression levels of the four genes. (e) The tSNE plots reveal NLRC4+ CASP1+ GSDMD+ cells and CASP4+ GSDMD+ cells in all cells. Red circles highlight the positive cell cluster. (f–j) Visualization and analysis of the GSE89567 dataset. All analysis and visualization methods are the same as those in (a–e). Statistics were calculated using two-tailed, unpaired Student's *t*-test with Welch's correction in (d, i). ns: not significant. ^∗∗^*p* < 0.01,  ^∗∗∗^*p* < 0.001, and^∗∗∗∗^*p* < 0.0001.

Other glioma scRNA-seq datasets were used to validate our above findings. First, we obtained the dataset from the CGGA database from Yu et al., including 6148 cells from 13 patients [27]. The cells were classified into 6 cell types, including BMDMs and microglia, by downscaling analysis and cell-type identification (Figures 8(a) and 8(b)). BMDMs had significantly higher expression levels of GSDMD, CASP1, CASP4, and NLRC4 than microglia (Figure 8(c)). In addition, we calculated the PyropScore for each cell using ssGSEA based on the expression levels of the four genes. The PyropScore of BMDMs was significantly higher than that of microglia (Figure 8(d)). The NLRC4+ CASP1+ GSDMD+ and CASP4+ GSDMD+ cell populations were also significantly enriched in the BMDM cell cluster (Figure 8(e)). Consistent results were obtained based on the analysis of another independent dataset, GSE89567, which contained 6341 cells from 10 patients [28] (Figures 8(f)–8(j)).

### 3.6. The Activation of Pathways of Response to LPS/Bacteria and Oxidative Stress in Pyroptotic BMDMs

**Figure 9:**
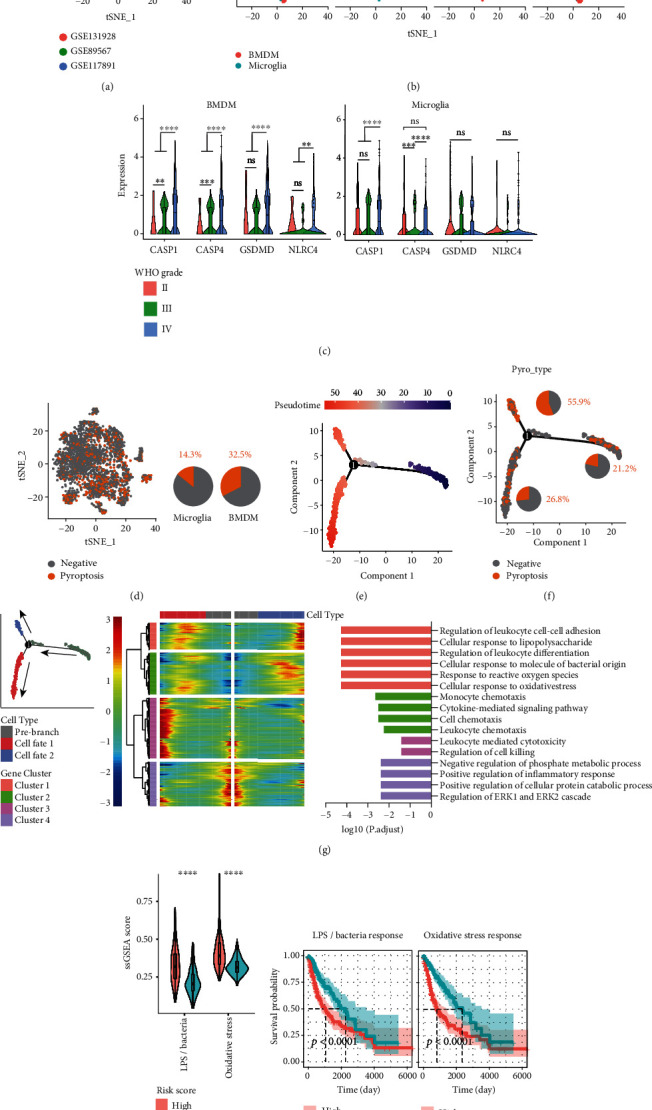
Activation of oxidative stress pathways in pyroptotic BMDMs was associated with poor prognosis. (a) BMDM and microglia identified in the GSE131928, GSE117891 and GSE117891 datasets were extracted and integrated using the harmony R package. (b) tSNE plots show the distribution of BMDMs and microglia in glioma samples of different disease grades after correction for batch effects. The pie charts show the percentage of BMDMs and microglia in each WHO grade of sample. (c) Comparison of CASP1, CASP4, GSDMD, and NLRC4 expression in BMDMs (left) and microglia (right) from samples of different WHO grades. (d) CASP1+ GSDMD+ and CASP4+ GSDMD+ cells were defined as pyroptotic cells, and the pyroptosis type of the cells was projected in the tSNE plot. The pie charts show the percentage of BMDMs and microglia in each WHO grade of sample. The pie chart shows the percentage of pyroptotic cells in BMDMs and microglia. (e, f) Trajectory of all BMDMs along pseudotime. The colors from blue to red represent the forward order of pseudotime. The pyroptosis type of the cells is projected on the trajectory. (g) Heatmap revealing the dynamic changes in gene expression during the differentiation process. From the middle to the left and to the right represent the process of changes in gene expression toward differentiation to the two cell fates. Differences in enriched pathways by GO between different phases (right panel). Genes were grouped into four clusters according to their expression patterns, and the results of GO enrichment analysis for each gene cluster are presented in different colors and shown below. (h, i) ssGSEA in TCGA cohort based on the LPS/bacterial response pathway and oxidative stress response pathway genes in Gene Cluster 1, comparing their differences between the high- and low-risk groups and their impact on overall survival. Statistics were calculated using the Wilcoxon test in (c). Statistics were calculated using two-tailed, unpaired Student's *t*-test with Welch's correction in (h). ns: not significant. ^∗∗^*p* < 0.01,  ^∗∗∗^*p* < 0.001, and^∗∗∗∗^*p* < 0.0001.

To further explore the differences between tumor-infiltrating BMDMs and microglia and the potential triggers of BMDM pyroptosis, the expression profiles of all BMDMs and microglia were extracted from the above 3 datasets, and the Harmony R package was used for batch effect correction. A total of 1423 BMDMs and 1441 microglia from 36 patients were finally integrated (Figure 9(a)). Notably, BMDM infiltration gradually replaced most microglia as disease grade progressed (Figure 9(b)), and BMDMs from high-grade gliomas had significantly higher expression of CASP1, CASP4, GSDMD, and NLRC4 genes than low-grade gliomas, whereas microglia did not have this conserved relationship (Figure 9(c)). This result is consistent with the previous results of a positive correlation between the Riskscore and BMDM infiltration obtained based on bulk RNA-seq analysis (Figure 6(e)). We defined CASP1+ GSDMD+ and CASP4+ GSDMD+ cells as pyroptotic cells and observed a significantly higher proportion of pyroptotic cells in BMDMs than in microglia (Figure 9(d)). To explore the characteristic changes during BMDM development toward the pyroptosis cell fate, we explored the BMDM differentiation trajectory using the Monocle R package (Figure 9(e)). Notably, pseudotime analysis revealed two cell fates of the BMDM developmental trajectory in gliomas, one of which had a significantly higher proportion of pyroptotic cells (Figure 9(f)). By integrating pathway enrichment and trajectory information, we found that BMDM development toward the pyroptosis cell fate was accompanied by the activation of the response pathway to lipopolysaccharide (LPS), bacteria and oxidative stress (Gene Cluster 1) (Figure 9(g)). LPS and bacterial infection (e.g., *Salmonella typhimurium*) are classic inducers of pyroptosis in macrophages [51], while oxidative stress has also been recently reported to lead to caspase1-GSDMD-mediated pyroptosis [52, 53]. Although the presence of LPS and bacterial infection in gliomas is less likely, aberrant activation of this response pathway and reactive oxygen species in the tumor microenvironment may lead to macrophage pyroptosis. In addition, we evaluated the activation of the LPS/bacteria response pathway and the oxidative stress response pathway in TCGA cohort using ssGSEA based on the corresponding genes in Gene Cluster 1. The results showed that the high-risk group had significantly higher LPS/bacteria response and oxidative stress response pathway scores (Figure 9(h)) and that high LPS/bacteria response pathway scores and oxidative stress pathway scores were associated with shorter overall survival (Figure 9(i)). The above results suggest that the activation of LPS/bacteria or oxidative stress pathways is associated with poor prognosis, suggesting that they may be triggers of BMDM pyroptosis, thereby affecting the tumor microenvironment and leading to tumor progression.

## 4. Discussion

Gliomas are the most common primary tumors of the central nervous system (CNS) and remain incurable, and a deeper understanding of their pathobiology is urgently needed [54]. The glioma tumor microenvironment has a large number of chemokines, cytokines, and growth factors. Despite the recruitment of a high abundance of infiltrating immune cells, such as microglia, peripheral macrophages, CD8+ T cells, CD4+ T cells, and Tregs, the chronic inflammatory environment leads to the establishment of a tumor immunosuppressive microenvironment, which ultimately promotes tumor development [55, 56].

Recently, an inflammatory cell death known as pyroptosis has emerged as an important mediator of the inflammatory response, and as research progresses, pyroptosis is being proven to be closely associated with an increasing number of types of inflammatory diseases and tumors [10, 57, 58]. Although several recent studies have expanded on the involvement of gasdermin family genes in pyroptosis pathways [59–61], the GSDMD-mediated pyroptosis signaling pathway triggered by inflammasomes has been shown to be the pathway most associated with the formation of an immunosuppressive microenvironment in a variety of tumors [62]. For example, in pancreatic ductal adenocarcinoma (PDA) and head and neck squamous cell carcinoma (HNSCC), inflammasomes of tumor-associated macrophages activate caspase-1 and mediate the cleavage of GSDMD and the release of mature IL1*β*, resulting in the suppression of CD8+ T cells [13, 14]. Although inflammasome-mediated pyroptosis in glioma has not been reported, IL1*β* has been shown to be a serum marker in glioblastoma [16, 17], which prompted us to explore potential pyroptosis pathways in glioma.

In this study, we conducted a comprehensive exploration of pyroptosis in glioma. We screened for prognosis-related genes at each key regulatory site of the pyroptosis pathway, obtained the NLRC4/CASP1/CASP4/GSDMD gene cluster, and developed a robust prognostic model based on this cluster. The differentially expressed genes that were upregulated in the high-risk group, defined by the expression of the four genes, were associated with the activation of multiple inflammatory response pathways and increased immune cell infiltration, which are typical results of pyroptosis. In addition, we demonstrated an immunosuppressive microenvironment in the high-risk group using multiple methods, including TIDE, ssGSEA, and landscape analysis of immune checkpoint expression profiles. Moreover, the differentially expressed genes that were in the high-risk group were strongly associated with synaptic establishment and synaptic signaling, suggesting that pyroptosis can lead to synaptic impairment and neurodegenerative diseases and may explain to some extent the clinical phenomenon of cognitive dysfunction associated with poor prognosis in glioblastoma patients [42]. Notably, the Riskscore was significantly positively correlated with BMDM infiltration, while the single-cell transcriptomics further demonstrated that NLRC4+ CASP1+ GSDMD+ and CASP4+ GSDMD+ cells were concentrated in a specific peripheral-derived BMDM cluster. Gene characteristics of this cell cluster were found to be associated with monocyte/leukocyte chemotaxis and the expression of IL1*β*, an important cytokine involved in the establishment of the immunosuppressive microenvironment, in several previous studies. More importantly, our analysis based on bulk RNA-seq datasets showed a significant positive correlation between pyroptosis gene expression and BMDM infiltration, and independent validation in multiple single-cell RNA-seq datasets further provided conclusive evidence that the four pyroptosis genes were highly expressed only in BMDMs and that pyroptosis gene expression levels were even higher in BMDMs from high-grade glioma samples. Finally, we revealed the activation of LPS/bacteria and oxidative stress response pathways during BMDM development toward the pyroptosis cell fate by pseudotime analysis, suggesting potential BMDM pyroptosis initiators. This is the first demonstration of a strong association between the pyroptosis signaling pathway and BMDM in glioma, providing novel insights into the pathological mechanisms of glioma.

Disulfiram (DSF), a recently demonstrated pyroptosis inhibitor [63], has been well validated in preclinical studies for the treatment of glioblastoma and has advanced to the clinical study phase as a novel adjuvant [64, 65]. In these studies, DSF was considered only as an acetaldehyde dehydrogenase (ALDH) inhibitor based on its classic function of treating alcohol addiction, but given its new status, the drug's function as an inhibitor of pyroptosis in glioma needs to be re-examined to guide the screening of suitable patients. In addition, our bulk RNA-seq-based analysis showed a significant positive correlation between the Riskscore and BMDM infiltration, and we further provided conclusive evidence in multiple independent single-cell RNA-seq datasets that four pyroptosis genes are highly expressed only in BMDMs. Several previous studies have provided solid evidence that the infiltration of BMDMs leads to tumor progression and the establishment of an immunosuppressive microenvironment [34, 66]. Thus, the model we developed can be used to predict the pyroptosis and BMDM infiltration levels in a patient's tumor microenvironment, thus assisting in the selection of candidate antipyroptosis drugs and antimacrophage drugs for the treatment of glioma. Therefore, this retrospective study is of great value, as it provides an in-depth exploration of glioma pathogenesis and its results suggest possibilities for drug development and repurposing based on the pyroptosis signaling pathway.

However, there are still some limitations: (1) the study was conducted based on retrospective data; thus, selection bias might be unavoidable, and (2) although we provided evidence based on bulk transcriptome, single-cell transcriptome and tissue microarray immunohistochemistry data demonstrating a strong relationship between glioma progression and pyroptosis, complex in vivo experiments, such as testing the rate of glioma tumorigenesis and immunosuppression of the tumor microenvironment in GSDMD-deficient mice, can provide more conclusive evidence for the value of pyroptosis as a drug target, which is a promising direction for subsequent studies.

## 5. Conclusions

Our study revealed a critical role of pyroptosis in maintaining immunosuppression in the tumor microenvironment and established a robust pyroptosis score as a prognostic biomarker. We further identified the pyroptosis BMDM cluster at single-cell resolution and preliminarily explored the trigger of BMDM pyroptosis, aberrant activation of pathways in response to LPS/bacteria and oxidative stress, providing potential targets for novel therapies against glioma, such as pyroptosis inhibitors and antimacrophage drugs.

## Figures and Tables

**Table 1:** Clinical characteristics of TCGA training cohort and CGGA validating cohort.

Characteristics	TCGA dataset					CGGA dataset				
	High risk (*N* = 298)	Low risk (*N* = 299)	Total (*N* = 597)	*p*	FDR	High risk (*N* = 157)	Low risk (*N* = 156)	Total (*N* = 313)	*p*	FDR
Histology				4.40*E* − 38	2.60*E* − 37				1.20*E* − 18	8.40*E* − 18
A	20 (3.35%)	33 (5.53%)	53 (8.88%)			16 (5.11%)	39 (12.46%)	55 (17.57%)		
AA	54 (9.05%)	56 (9.38%)	110 (18.43%)			33 (10.54%)	24 (7.67%)	57 (18.21%)		
AO	28 (4.69%)	57 (9.55%)	85 (14.24%)			0 (0.0*e* + 0%)	12 (3.83%)	12 (3.83%)		
AOA	16 (2.68%)	23 (3.85%)	39 (6.53%)			—	—	—		
GBM	145 (24.29%)	7 (1.17%)	152 (25.46%)			102 (32.59%)	35 (11.18%)	137 (43.77%)		
O	24 (4.02%)	90 (15.08%)	114 (19.10%)			3 (0.96%)	45 (14.38%)	48 (15.34%)		
OA	11 (1.84%)	33 (5.53%)	44 (7.37%)			—	—	—		
Grade				9.00*E* − 40	6.30*E* − 39	18 (5.75%)	80 (25.56%)	98 (31.31%)	7.70*E* − 16	4.60*E* − 15
WHO II	55 (9.21%)	156 (26.13%)	211 (35.34%)			34 (10.86%)	40 (12.78%)	74 (23.64%)		
WHO III	98 (16.42%)	136 (22.78%)	234 (39.20%)			102 (32.59%)	35 (11.18%)	137 (43.77%)		
WHO IV	145 (24.29%)	7 (1.17%)	152 (25.46%)							
Gender				0.97	0.97					
Female	126 (21.11%)	125 (20.94%)	251 (42.04%)			55 (17.57%)	61 (19.49%)	116 (37.06%)	0.53	0.6
Male	172 (28.81%)	174 (29.15%)	346 (57.96%)			102 (32.59%)	95 (30.35%)	197 (62.94%)		
Age										
Mean ± SD	52.36 ± 15.86	42.40 ± 13.04	47.37 ± 15.34			47.03 ± 12.78	39.28 ± 9.71	43.17 ± 11.98		
OS										
Mean ± SD	524.51 ± 715.63	775.33 ± 915.69	650.13 ± 830.79			741.79 ± 905.26	2164.90 ± 1586.83	1451.07 ± 1472.63		
Censor				3.50*E* − 13	1.40*E* − 12				1.30*E* − 10	5.10*E* − 10
Alive	168 (28.14%)	251 (42.04%)	419 (70.18%)			21 (6.71%)	74 (23.64%)	95 (30.35%)		
Dead	130 (21.78%)	48 (8.04%)	178 (29.82%)			136 (43.45%)	82 (26.20%)	218 (69.65%)		
Temozolomide				5.90*E* − 02	0.2				0.2	0.6
No	120 (20.10%)	155 (25.96%)	275 (46.06%)			48 (15.34%)	62 (19.81%)	110 (35.14%)		
Yes	178 (29.82%)	144 (24.12%)	322 (53.94%)			103 (32.91%)	87 (27.80%)	190 (60.70%)		
Chemotherapy				0.14	0.28				0.2	0.6
No	107 (17.92%)	126 (21.11%)	233 (39.03%)			48 (15.34%)	62 (19.81%)	110 (35.14%)		
Yes	191 (31.99%)	173 (28.98%)	364 (60.97%)			103 (32.91%)	87 (27.80%)	190 (60.70%)		
IDH mutation				3.00*E* − 50	2.40*E* − 49				1.20*E* − 23	2.80*E* − 22
Mutant	94 (15.75%)	274 (45.90%)	368 (61.64%)			39 (12.46%)	128 (40.89%)	167 (53.35%)		
Wild type	199 (33.33%)	24 (4.02%)	223 (37.35%)			118 (37.70%)	27 (8.63%)	145 (46.33%)		
1p19q codeletion				1.10*E* − 13	5.30*E* − 13				1.50*E* − 13	9.20*E* − 13
Codel	33 (5.53%)	114 (19.10%)	147 (24.62%)			4 (1.28%)	58 (18.53%)	62 (19.81%)		
Noncodel	261 (43.72%)	184 (30.82%)	445 (74.54%)			148 (47.28%)	95 (30.35%)	243 (77.64%)		

## Data Availability

The datasets analyzed during the current study are available in TCGA database (https://portal.gdc.cancer.gov/), CGGA database (http://www.cgga.org.cn/), GEO database (https://www.ncbi.nlm.nih.gov/geo/), and Single Cell Portal database (https://singlecell.broadinstitute.org/single_cell).

## Abbreviations

CNS: Central nervous system
ESTIMATE: Estimation of STromal and immune cells in MAlignant tumor tissues using Expression data
TIDE: Tumor Immune Dysfunction and Exclusion
ssGSEA: Single-sample gene set enrichment analysis
TME: Tumor microenvironment
scRNA-seq: Single-cell RNA sequencing
BMDM: Bone marrow-derived macrophages
GBM: Glioblastoma
LGG: Low-grade glioma
WHO: The World Health Organization
IDH: Isocitrate dehydrogenase
TCGA: The Cancer Genome Atlas
CGGA: Chinese Glioma Genome Atlas
GEO: Gene-expression omnibus
DEGs: Differentially expressed genes
Log2FC: Log2 fold change
CNV: Copy number variation
TMB: Tumor mutational burden
ICB: Immune checkpoint blockade
DSF: Disulfiram
GO: Gene Ontology analysis
KEGG: Kyoto Encyclopedia of Genes and Genomes.

## Supplementary Materials

Supplementary Materials Supplementary Figure S1: patients with IDH mutations have a shorter overall survival. Supplementary Figure S2: CASP1 and CASP4 were positively correlated with GSDMD expression in different datasets. Supplementary Figure S3: the inflammasome gene NLRC4 was associated with prognosis in different datasets. Supplementary Figure S4: the NLRC4/CASP1/CASP4/GSDMD gene clusters have highly conserved positive correlation expression pattern. Supplementary Figure S5: performance of the prognostic model developed in this study compared with the established models. Supplementary Figure S6: CNV and TMB differences between the high-and low-risk groups. Supplementary Figure S7: drug sensitivity analysis based on the four pyroptosis genes. Supplementary Figure S8: BMDM infiltration has a robust correlation with glioma prognosis, Riskscore, and M2 macrophage infiltration. Supplementary Figure S9: macrophage clusters were clustered into BMDM and microglia subpopulations by ssGSEA. Supplementary Table S1: gene sets for ssGSEA to estimate tumor microenvironment inhibitory degree. Supplementary Table S2: gene sets from Robert B for ssGSEA to estimate BMDM and microglia infiltration. Supplementary Table S3: gene sets from Sören M for ssGSEA to estimate BMDM and microglia infiltration.

## References

[1] Yang K., Wu Z., Zhang H. (2022). Glioma targeted therapy: insight into future of molecular approaches. *Molecular Cancer*.

[2] Louis D. N., Perry A., Wesseling P. (2021). The 2021 WHO classification of tumors of the central nervous system: a summary. *Neuro-Oncology*.

[3] Han S., Liu Y., Cai S. R. J. (2020). IDH mutation in glioma: molecular mechanisms and potential therapeutic targets. *British Journal of Cancer*.

[4] Weller M., Stupp R., Reifenberger G. (2010). MGMT promoter methylation in malignant gliomas: ready for personalized medicine?. *Nature Reviews. Neurology*.

[5] Shi J., Zhao Y., Wang K. (2015). Cleavage of GSDMD by inflammatory caspases determines pyroptotic cell death. *Nature*.

[6] Wang Y., Gao W., Shi X. (2017). Chemotherapy drugs induce pyroptosis through caspase-3 cleavage of a gasdermin. *Nature*.

[7] Yu P., Zhang X., Liu N., Tang L., Peng C., Chen X. (2021). Pyroptosis: mechanisms and diseases. *Signal Transduction and Targeted Therapy*.

[8] Jorgensen I., Rayamajhi M., Miao E. A. (2017). Programmed cell death as a defence against infection. *Nature Reviews Immunology*.

[9] Tan Y., Chen Q., Li X. (2021). Pyroptosis: a new paradigm of cell death for fighting against cancer. *Journal of Experimental & Clinical Cancer Research*.

[10] Wu L., Li L., Li S. (2022). Macrophage-mediated tumor-targeted delivery of engineered Salmonella typhimurium VNP20009 in anti-PD1 therapy against melanoma. *Acta Pharmaceutica Sinica B*.

[11] Xia X., Wang X., Cheng Z. (2019). The role of pyroptosis in cancer: pro-cancer or pro"host"?. *Cell Death & Disease*.

[12] Evavold C. L., Ruan J., Tan Y., Xia S., Wu H., Kagan J. C. (2018). The pore-forming protein Gasdermin D regulates Interleukin-1 secretion from living macrophages. *Immunity*.

[13] Chen L., Huang C. F., Li Y. C. (2018). Blockage of the NLRP3 inflammasome by MCC950 improves anti-tumor immune responses in head and neck squamous cell carcinoma. *Cellular and Molecular Life Sciences*.

[14] Daley D., Mani V. R., Mohan N. (2017). NLRP3 signaling drives macrophage-induced adaptive immune suppression in pancreatic carcinoma. *The Journal of Experimental Medicine*.

[15] Kaplanov I., Carmi Y., Kornetsky R. (2019). Blocking IL-1*β* reverses the immunosuppression in mouse breast cancer and synergizes with anti-PD-1 for tumor abrogation. *Proceedings of the National Academy of Sciences of the United States of America*.

[16] Gao Y., Zhang E., Liu B. (2019). Integrated analysis identified core signal pathways and hypoxic characteristics of human glioblastoma. *Journal of Cellular and Molecular Medicine*.

[17] Tarassishin L., Casper D., Lee S. C. (2014). Aberrant expression of interleukin-1*β* and inflammasome activation in human malignant gliomas. *PLoS One*.

[18] Chen Z., Giotti B., Kaluzova M. (2022). A paracrine circuit of IL-1*β*/IL-1R1 between myeloid and tumor cells drives glioblastoma progression. *bioRxiv*.

[19] Shen H., Han C., Yang Y. (2021). Pyroptosis executive protein GSDMD as a biomarker for diagnosis and identification of Alzheimer's disease. *Brain and Behavior*.

[20] Humphries F., Shmuel-Galia L., Ketelut-Carneiro N. (2020). Succination inactivates gasdermin D and blocks pyroptosis. *Science*.

[21] Kim H., Seo J. S., Lee S. Y. (2020). AIM2 inflammasome contributes to brain injury and chronic post-stroke cognitive impairment in mice. *Brain, Behavior, and Immunity*.

[22] Zhang M., Cheng Y., Xue Z., Sun Q., Zhang J. (2021). A novel pyroptosis-related gene signature predicts the prognosis of glioma through immune infiltration. *BMC Cancer*.

[23] Chao B., Jiang F., Bai H., Meng P., Wang L., Wang F. (2022). Predicting the prognosis of glioma by pyroptosis-related signature. *Journal of Cellular and Molecular Medicine*.

[24] Bao Z. S., Chen H. M., Yang M. Y. (2014). RNA-seq of 272 gliomas revealed a novel, recurrent PTPRZ1-MET fusion transcript in secondary glioblastomas. *Genome Research*.

[25] Gravendeel L. A., Kouwenhoven M. C., Gevaert O. (2010). Intrinsic gene expression profiles of gliomas are a better predictor of survival than histology. *Cancer Research*.

[26] Neftel C., Laffy J., Filbin M. G. (2019). An integrative model of cellular states, plasticity, and genetics for glioblastoma. *Cell*.

[27] Yu K., Hu Y. Q., Wu F. (2020). Surveying brain tumor heterogeneity by single-cell RNA-sequencing of multi-sector biopsies. *National Science Review*.

[28] Venteicher A. S., Tirosh I., Hebert C. (2017). Decoupling genetics, lineages, and microenvironment in IDH-mutant gliomas by single-cell RNA-seq. *Science*.

[29] Yoshihara K., Shahmoradgoli M., Martínez E. (2013). Inferring tumour purity and stromal and immune cell admixture from expression data. *Nature Communications*.

[30] Newman A. M., Liu C. L., Green M. R. (2015). Robust enumeration of cell subsets from tissue expression profiles. *Nature Methods*.

[31] Jiang P., Gu S., Pan D. (2018). Signatures of T cell dysfunction and exclusion predict cancer immunotherapy response. *Nature Medicine*.

[32] Mariathasan S., Turley S. J., Nickles D. (2018). TGF*β* attenuates tumour response to PD-L1 blockade by contributing to exclusion of T cells. *Nature*.

[33] Wu L., Zhou F., Xin W. (2022). MAGP2 induces tumor progression by enhancing uPAR-mediated cell proliferation. *Cellular Signalling*.

[34] Bowman R. L., Klemm F., Akkari L. (2016). Macrophage ontogeny underlies differences in tumor-specific education in brain malignancies. *Cell Reports*.

[35] Müller S., Kohanbash G., Liu S. J. (2017). Single-cell profiling of human gliomas reveals macrophage ontogeny as a basis for regional differences in macrophage activation in the tumor microenvironment. *Genome Biology*.

[36] Li X., Garg M., Jia T. (2022). Single-cell analysis reveals the immune characteristics of myeloid cells and memory T cells in recovered COVID-19 patients with different severities. *Frontiers in Immunology*.

[37] Korsunsky I., Millard N., Fan J. (2019). Fast, sensitive and accurate integration of single-cell data with harmony. *Nature Methods*.

[38] Sun D., Guan X., Moran A. E. (2021). Identifying phenotype-associated subpopulations by integrating bulk and single-cell sequencing data. *Nature Biotechnology*.

[39] He W. T., Wan H. Q., Hu L. C. (2015). Gasdermin D is an executor of pyroptosis and required for interleukin-1*β* secretion. *Cell Research*.

[40] Liu J., Gao L., Zhu X. (2021). Gasdermin D is a novel prognostic biomarker and relates to TMZ response in glioblastoma. *Cancers*.

[41] Sollberger G., Strittmatter G. E., Garstkiewicz M., Sand J., Beer H. D. (2014). Caspase-1: the inflammasome and beyond. *Innate Immunity*.

[42] Bruhn H., Blystad I., Milos P. (2022). Initial cognitive impairment predicts shorter survival of patients with glioblastoma. *Acta Neurologica Scandinavica*.

[43] Burke T. P., Engström P., Chavez R. A., Fonbuena J. A., Vance R. E., Welch M. D. (2020). "Inflammasome-mediated antagonism of type I interferon enhances Rickettsia pathogenesis," nature. *Microbiology*.

[44] Haase S., Garcia-Fabiani M. B., Carney S. (2018). Mutant ATRX: uncovering a new therapeutic target for glioma. *Expert Opinion on Therapeutic Targets*.

[45] Benitez J. A., Ma J., Antonio M. (2017). PTEN regulates glioblastoma oncogenesis through chromatin-associated complexes of DAXX and histone H3.3. *Nature Communications*.

[46] Zhang H., Luo Y. B., Wu W. T. (2021). The molecular feature of macrophages in tumor immune microenvironment of glioma patients. *Computational and Structural Biotechnology Journal*.

[47] Zhang N., Zhang H., Wang Z. (2021). Immune infiltrating cells-derived risk signature based on large-scale analysis defines immune landscape and predicts immunotherapy responses in glioma tumor microenvironment. *Frontiers in Immunology*.

[48] Chinen T., Kannan A. K., Levine A. G. (2016). An essential role for the IL-2 receptor in T_reg_ cell function. *Nature Immunology*.

[49] Wang S. M., Lin H. Y., Chen Y. L. (2019). CCAAT/enhancer-binding protein delta regulates the stemness of glioma stem-like cells through activating PDGFA expression upon inflammatory stimulation. *Journal of Neuroinflammation*.

[50] Kunze R., Urrutia A., Hoffmann A. (2015). Dimethyl fumarate attenuates cerebral edema formation by protecting the blood- brain barrier integrity. *Experimental Neurology*.

[51] Wu L., Bao F., Li L., Yin X., Hua Z. (2022). Bacterially mediated drug delivery and therapeutics: strategies and advancements. *Advanced Drug Delivery Reviews*.

[52] Li H. B., Xia Z. Y., Chen Y. F., Qi D., Zheng H. (2018). Mechanism and therapies of oxidative stress-mediated cell death in ischemia reperfusion injury. *Oxidative Medicine and Cellular Longevity*.

[53] Wang S., Ji L. Y., Li L., Li J. M. (2019). Oxidative stress, autophagy and pyroptosis in the neovascularization of oxygen-induced retinopathy in mice. *Molecular Medicine Reports*.

[54] Davis M. E. (2016). Glioblastoma: overview of disease and treatment. *Clinical Journal of Oncology Nursing*.

[55] Gieryng A., Pszczolkowska D., Walentynowicz K. A., Rajan W. D., Kaminska B. (2017). Immune microenvironment of gliomas. *Laboratory Investigation*.

[56] Zhang H., Wang Z., Dai Z. (2021). Novel immune infiltrating cell signature based on cell pair algorithm is a prognostic marker in cancer. *Frontiers in Immunology*.

[57] Zheng X., Chen W., Gong F., Chen Y., Chen E. (2021). The role and mechanism of pyroptosis and potential therapeutic targets in sepsis: a review. *Frontiers in Immunology*.

[58] Liu P., Zhang Z., Li Y. (2021). Relevance of the pyroptosis-related inflammasome pathway in the pathogenesis of diabetic kidney disease. *Frontiers in Immunology*.

[59] Deng W., Bai Y., Deng F. (2022). Streptococcal pyrogenic exotoxin B cleaves GSDMA and triggers pyroptosis. *Nature*.

[60] Hou J., Zhao R., Xia W. (2020). PD-L1-mediated gasdermin C expression switches apoptosis to pyroptosis in cancer cells and facilitates tumour necrosis. *Nature Cell Biology*.

[61] Zhou Z., He H., Wang K. (2020). Granzyme A from cytotoxic lymphocytes cleaves GSDMB to trigger pyroptosis in target cells. *Science*.

[62] Burdette B. E., Esparza A. N., Zhu H., Wang S. (2021). Gasdermin D in pyroptosis. *Acta Pharmaceutica Sinica B*.

[63] Hu J. J., Liu X., Xia S. (2020). FDA-approved disulfiram inhibits pyroptosis by blocking gasdermin D pore formation. *Nature Immunology*.

[64] Liu C. C., Wu C. L., Lin M. X., Sze C. I., Gean P. W. (2021). Disulfiram sensitizes a therapeutic-resistant glioblastoma to the TGF-beta receptor inhibitor. *International Journal of Molecular Sciences*.

[65] Lu C., Li X. Y., Ren Y. Y., Zhang X. (2021). Disulfiram: a novel repurposed drug for cancer therapy. *Cancer Chemotherapy and Pharmacology*.

[66] Pinton L., Masetto E., Vettore M. (2019). The immune suppressive microenvironment of human gliomas depends on the accumulation of bone marrow-derived macrophages in the center of the lesion. *Journal for Immunotherapy of Cancer*.

