# Supplementary material for: Bulk and Single-Cell Transcriptome Analyses Revealed That the Pyroptosis of Glioma-Associated Macrophages Participates in Tumor Progression and Immunosuppression
Source: Oxid Med Cell Longev. 2022 Sep 26;2022:1803544. doi: 10.1155/2022/1803544 (PMC9529448; doi:10.1155/2022/1803544)
Supplement: Supplementary Materials — Supplementary Figure S1: patients with IDH mutations have a shorter overall survival. Supplementary Figure S2: CASP1 and CASP4 were positively correlated with GSDMD expression in different datasets. Supplementary Figure S3: the inflammasome gene NLRC4 was associated with prognosis in different datasets. Supplementary Figure S4: the NLRC4/CASP1/CASP4/GSDMD gene clusters have highly conserved positive correlation expression pattern. Supplementary Figure S5: performance of the prognostic model developed in this study compared with the established models. Supplementary Figure S6: CNV and TMB differences between the high-and low-risk groups. Supplementary Figure S7: drug sensitivity analysis based on the four pyroptosis genes. Supplementary Figure S8: BMDM infiltration has a robust correlation with glioma prognosis, Riskscore, and M2 macrophage infiltration. Supplementary Figure S9: macrophage clusters were clustered into BMDM and microglia subpopulations by ssGSEA. Supplementary Table S1: gene sets for ssGSEA to estimate tumor microenvironment inhibitory degree. Supplementary Table S2: gene sets from Robert B for ssGSEA to estimate BMDM and microglia infiltration. Supplementary Table S3: gene sets from Sören M for ssGSEA to estimate BMDM and microglia infiltration. [file 1803544.f1.docx]

Supplementary Material


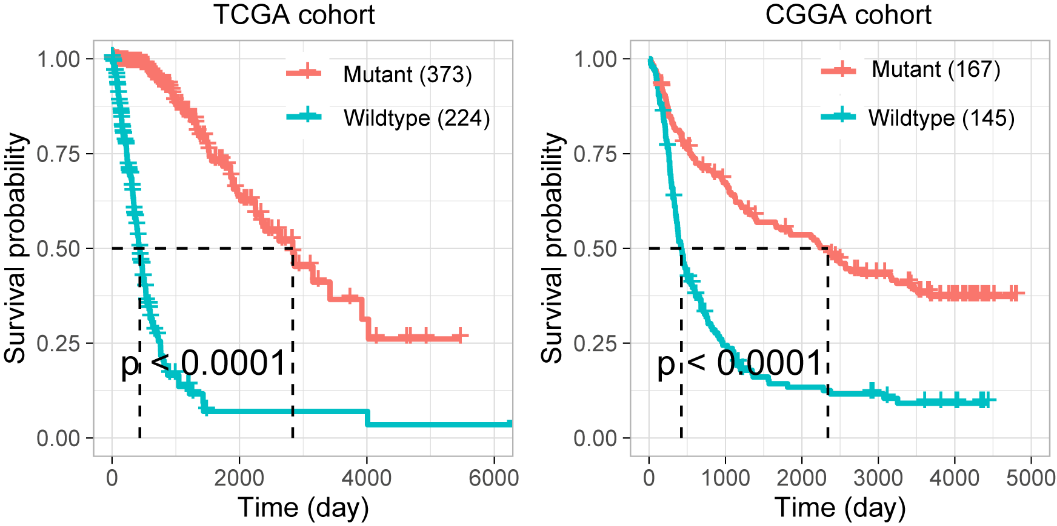


**Supplementary Figure. S1 Patients with IDH mutations have a shorter overall survival.** Kaplan-Meier curves were used to analyze the relationship between IDH mutation status and overall survival (OS) in the TCGA dataset and CGGA dataset.


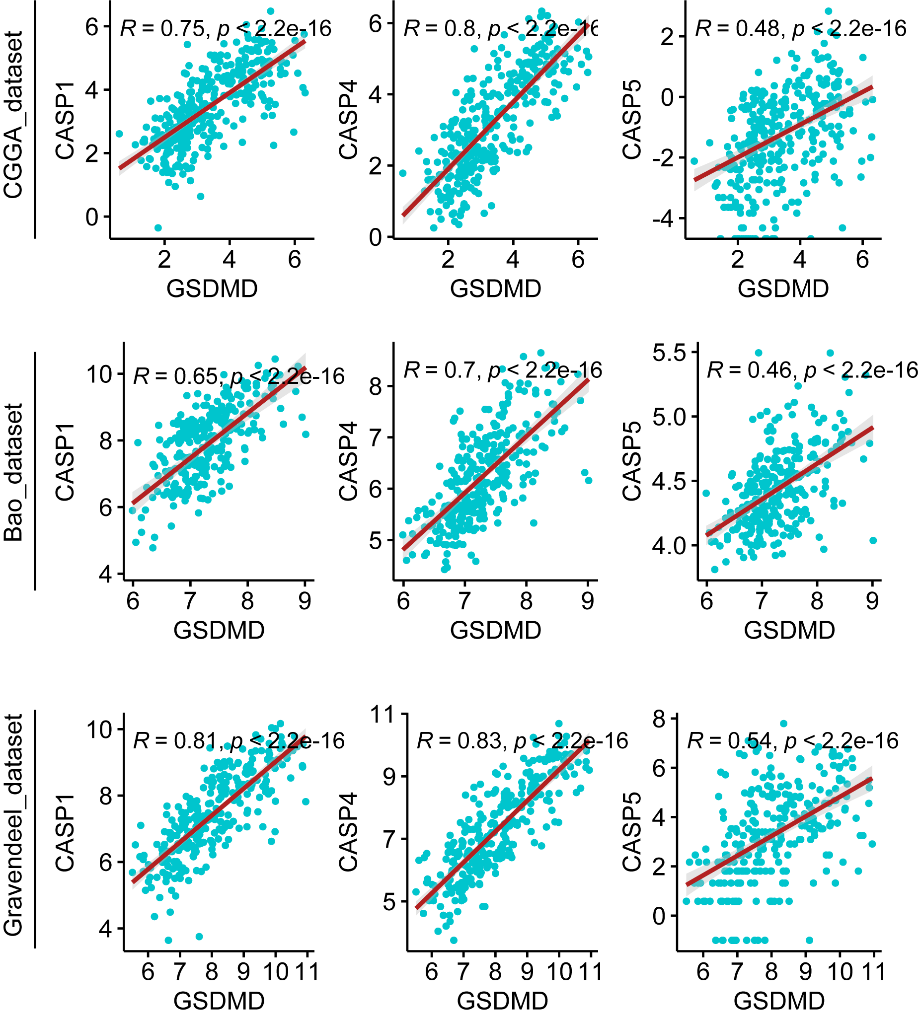


**Supplementary Figure. S2 CASP1/CASP4 were highly conserved and positively correlated with GSDMD expression in different datasets.** Scatter plots of the correlation between GSDMD and CASP1/CASP4/CASP5 gene expression in CGGA dataset, Bao dataset (GSE48865) and Gravendeel dataset (GSE12907, GSE4271). Spearman coefficient was used to characterize the degree of positive correlation.


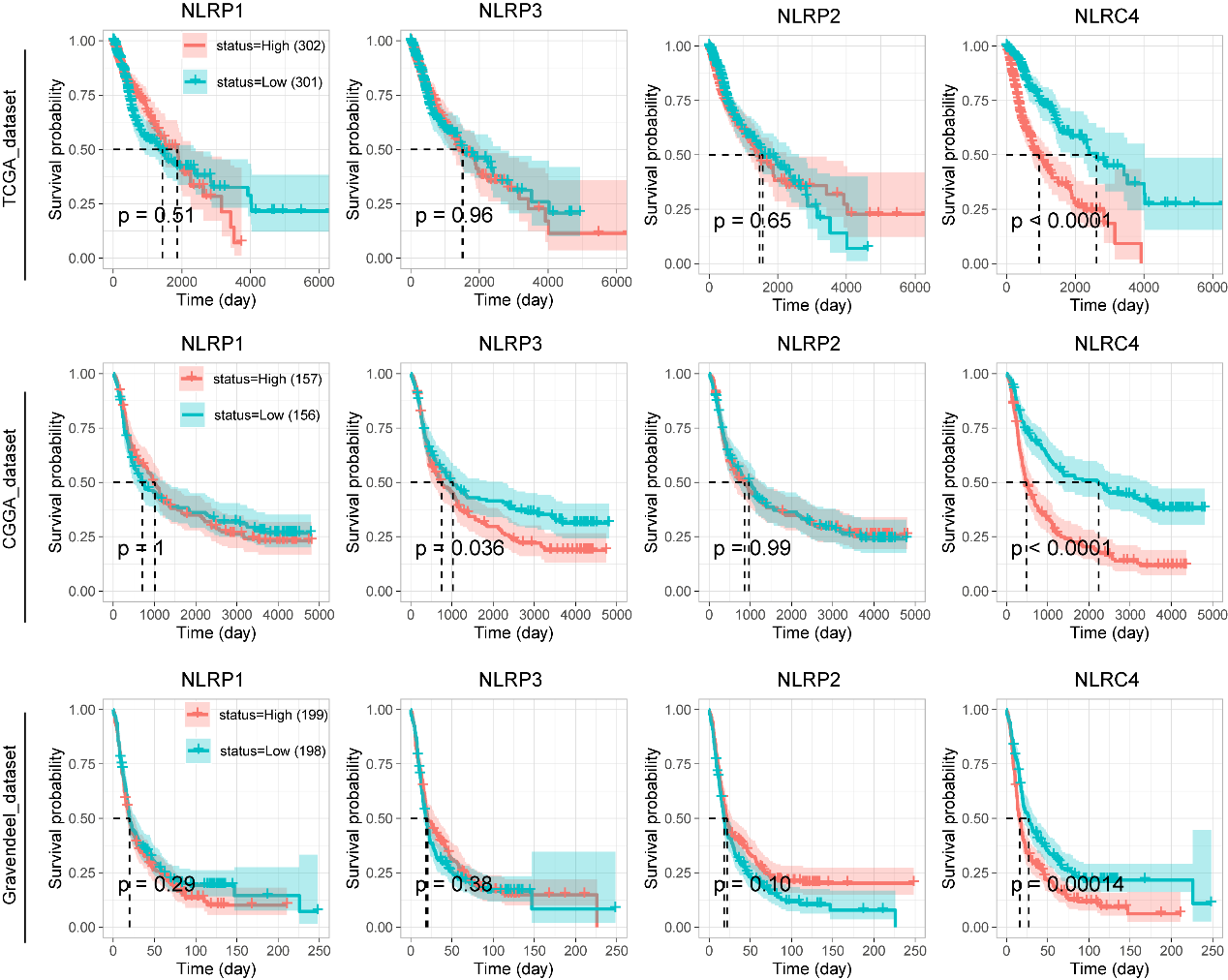


**Supplementary Figure. S3 The inflammasome gene NLRC4 was associated with prognosis in different datasets.** Kaplan-Meier curves were used to analyze the relationship between gene expression levels of inflammasome genes and overall survival (OS) in the TCGA dataset, CGGA dataset and Gravendeel dataset (GSE12907, GSE4271). The median expression value of each gene was used to classify the high and low expression groups.


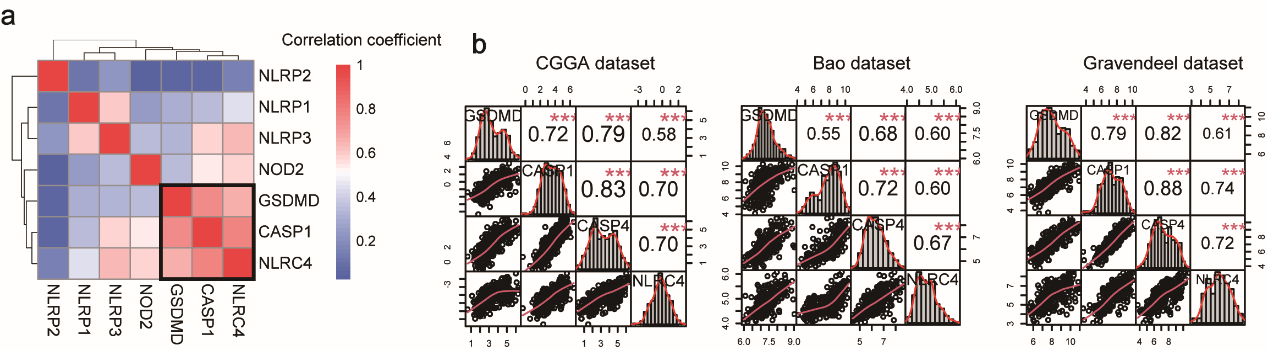


**Supplementary Figure. S4 The NLRC4/CASP1/CASP4/GSDMD gene cluster showed a highly conserved positive correlation expression pattern in multiple independent datasets.** The PerformanceAnalytics R package was used to plot the expression correlation matrix and calculate the correlation coefficients for the NLRC4/CASP1/CASP4/GSDMD gene clusters in the CGGA dataset, the Bao dataset (GSE48865) and the Gravendeel dataset (GSE12907, GSE4271). The numbers in the matrix indicate the correlation coefficients. ****p* < 0.001


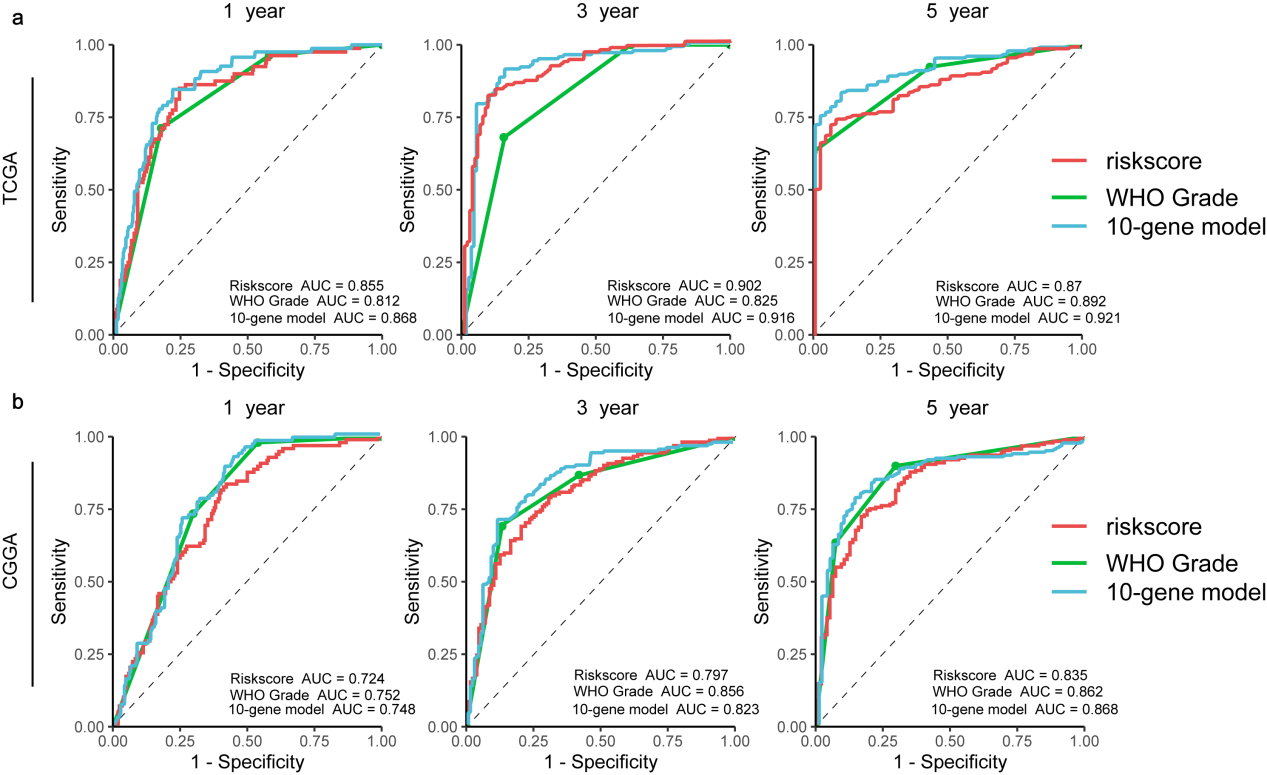


**Supplementary Figure. S5 The prognostic model has the ability to predict overall survival approximating the WHO grading system and previously established 10-pyroptosis-gene prognostic model.** The predictive ability of Riskscore compared to WHO grading system and previously established 10-pyroptosis-gene prognostic model in terms of 1-year AUC, 3-year AUC, and 5-year AUC was verified by ROC curve analysis in the TCGA cohort (**a**) and CGGA cohort (**b**).


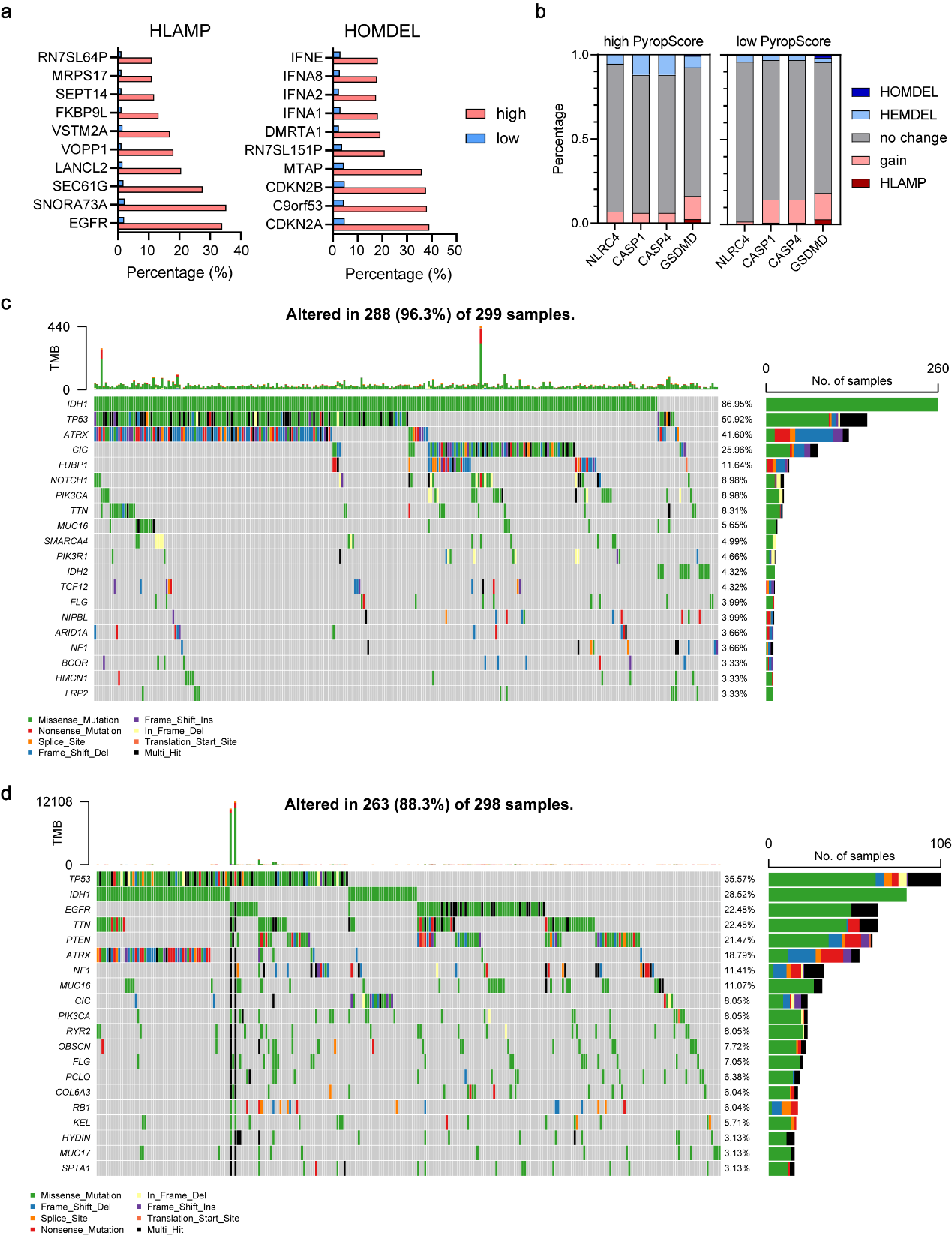


**Supplementary Figure. S6 Copy number variation (CNV) and tumor mutational burden (TMB) differences between high and low risk groups.** (**a**) High level amplification genes (HLAMP) and homozygous deletion genes (HOMDEL) with the largest difference in the proportion between high and low risk groups. (**b**) CNV profile of four pyroptosis death genes in high- and low-risk groups. HOMDEL, homozygous deletion; HEMDEL, hemizygous deletion; HLAMP, high level amplification. (**c**) Mutation profiles of samples from low-risk groups visualized using waterfall plots. (**d**) Mutation profiles of samples from high-risk groups visualized using waterfall plots.
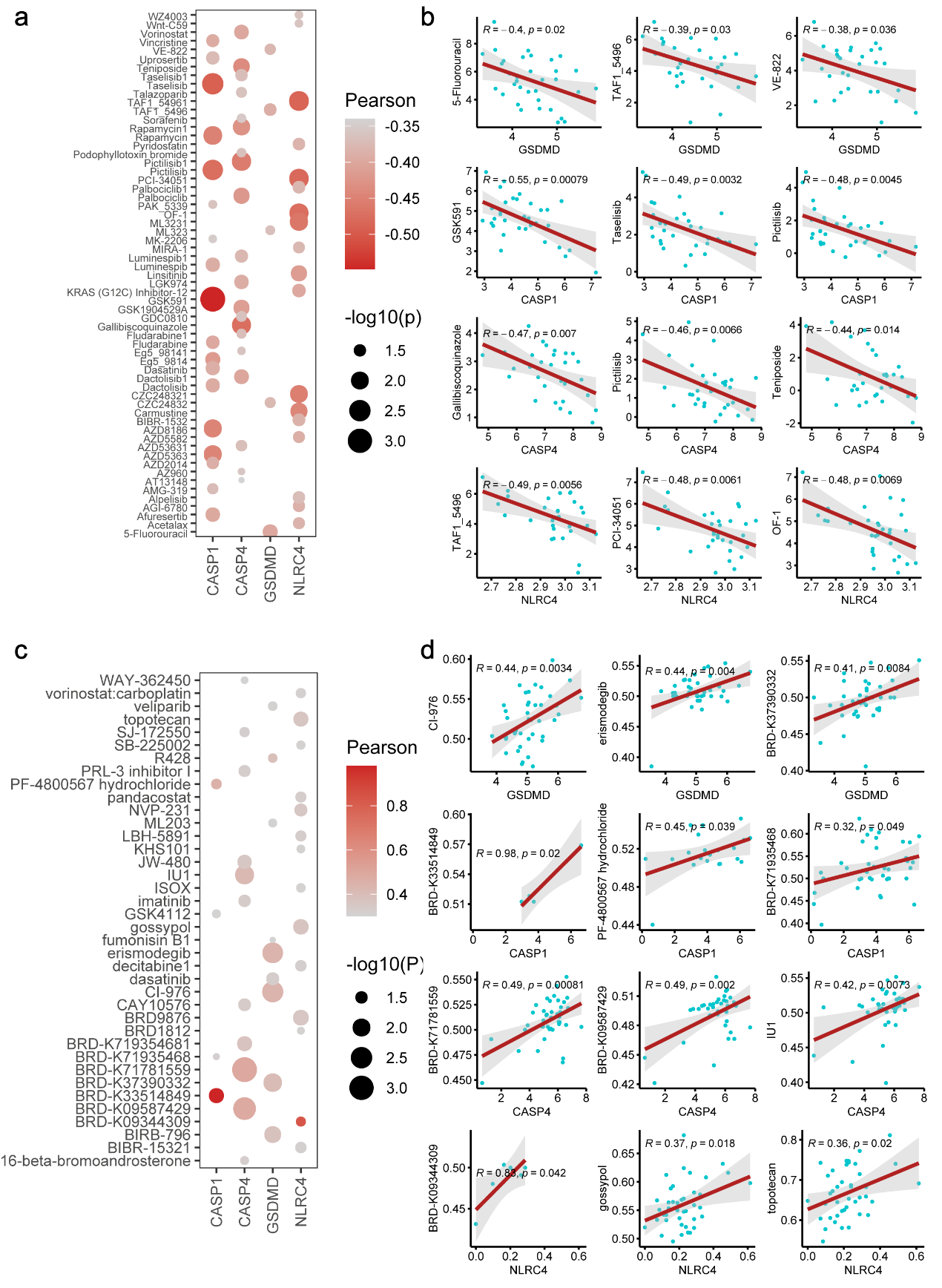


**Supplementary Figure. S7 Drug sensitivity analysis based on the four pyroptosis genes.** (**a, b**) Bubble plots of correlation analysis between the expression of focal death genes and IC50 for glioma cell lines in the GDSC database, and scatter plots of the three representative drugs with the highest Pearson correlation coefficients corresponding to each gene were shown in **b**. The x-axis indicates the gene expression and the y-axis indicates the IC50 after different drug treatments, and the negative correlation between the two indicates that drug sensitivity is positively correlated with gene expression. (**c, d**) Bubble plots of correlation analysis between the expression of focal death genes and IC50 for glioma cell lines in the CTRP database, and scatter plots of the three representative drugs with the highest Pearson correlation coefficients corresponding to each gene were shown in **b**. The x-axis indicates the gene expression and the y-axis indicates the calculated drug sensitivity (1-(AUC/30)), and the positive correlation between the two indicates that drug sensitivity is positively correlated with gene expression.


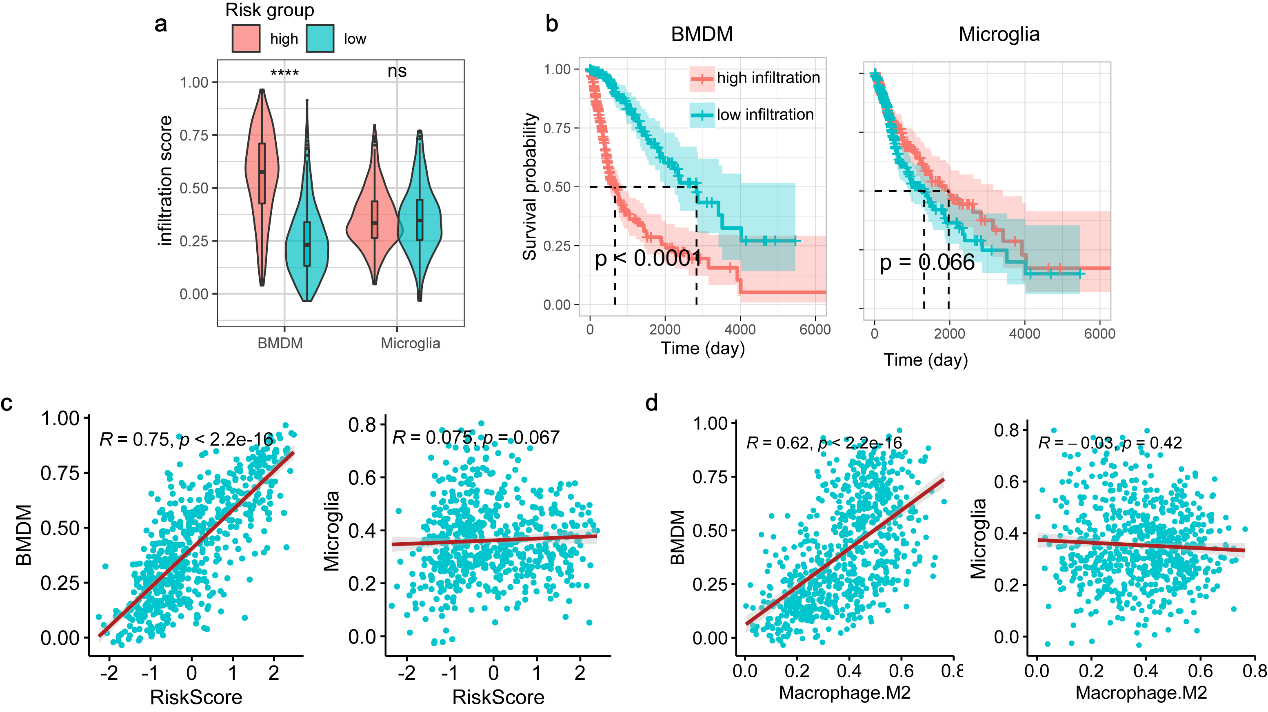


**Supplementary Figure. S8 BMDM infiltration has a robust correlation with glioma prognosis, Riskscore and M2 macrophage infiltration.** The infiltration scores of BMDM and microglia were predicted based on the differentially expressed genes of BMDM and microglia demonstrated by Sören M et al. and used in the following analyses. (**a**) Comparison of infiltration scores of BMDM and microglia in high- and low-risk groups. (**b**) Kaplan-Meier curves were used to analyze the relationship between BMDM and microglia infiltration and overall survival (OS) in the TCGA cohort. The median infiltration score was used to classify the high- and low-infiltration groups. (**c**) Scatter plots of the correlation between Riskscore and BMDM infiltration or microglia in TCGA cohort. Spearman coefficient was used to characterize the degree of positive correlation. (**d**) Scatter plots of the correlation between M2 infiltration and BMDM or microglia infiltration in TCGA cohort. Spearman coefficient was used to characterize the degree of positive correlation. Statistics were calculated using the two-tailed, unpaired Student’s t test with Welch’s correction in **a**. ns = not significant, *****p* < 0.0001.


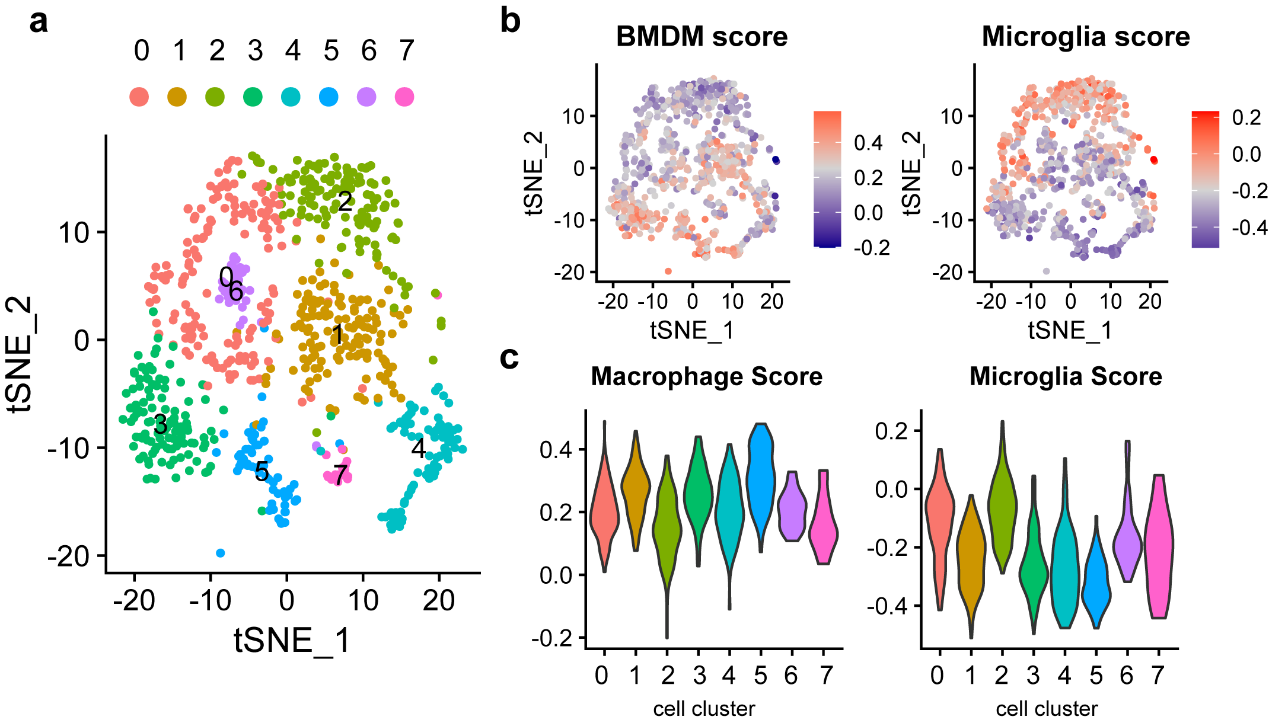


**Supplementary Figure. S9 Macrophage clusters were clustered into BMDM and microglia subpopulations by ssGSEA.** (**a**) tSNE plot of all macrophage cells by cell cluster. (**b**) Prediction of Macrophage Score and Microglia Score for each cell based on differentially expressed genes (DEGs) between microglia and bone marrow-derived macrophages (BMDM) using ssGSEA, and mapping the results on tSNE plot. (**c**) Violin plots of Macrophage Score and Microglia Score for different macrophage clusters.

**Supplementary Table. S1 Gene sets for ssGSEA to estimate tumor microenvironment inhibitory degree**

| **Gene Signature** | **Genes for ssGSEA** |
| --- | --- |
| **TGF-β response scores (TBRS)** | ACTA2, ACTG2, ADAM12, ADAM19, CNN1, COL4A1, CTGF, CTPS1, FAM101B, FSTL3, HSPB1, IGFBP3, PXDC1, SEMA7A, SH3PXD2A, TAGLN, TGFBI, TNS1, TPM1 |
| **Immune checkpoint** | CD274, CTLA4, HAVCR2, LAG3, PDCD1, PDCD1LG2, TIGIT |

**Supplementary Table. S2 Gene sets from Robert B for ssGSEA to estimate BMDM and microglia infiltration**

| **Cluster** | **Genes for ssGSEA** |
| --- | --- |
| **BMDM** | ARSI, SIRPB1A, KLRI2, GRAP2, ZFP366, LAD1, MAPK13, GPR141, IL2RA, KLRA2, RAB38, KLRK1, OCSTAMP, CD209A, AMICA1, ART2A-PS, HIC1, GAPT, ATP8B4, STAP1, SPINT2, CCL22, BTLA, PLBD1, SIRPB1B, KLRD1, DPP4, TCEA3, INPP5J, HEPACAM2, CLEC12A, KMO, NAPSA, IFI203, ITGB7, GM5150 |
| **Microglia** | 2010001M06RIK, NAALAD2, TPPP, 1190007F08RIK, HSPB3, SLC2A5, LOX, THRSP, SALL1, FAM176A, CSMD3, LIPH, ECSCR, MLPH, RIC3, TLN2, SPARC, CST7, AK1, TOM1L1, CRYBB1, TMEM100, SGCE, TMEM8C, NAV3, MASP1, MYADML2, GPR84, DUSP27, PROX1, MRC2, ZFP354A, COL6A3, PMP22, TSPAN7, GPR56 |

**Supplementary Table. S3 Gene sets from Sören M for ssGSEA to estimate BMDM and microglia infiltration**

| **Cluster** | **Genes for ssGSEA** |
| --- | --- |
| **BMDM** | CD163, HLA-DQA1, FCGBP, PLTP, TMEM176B, PLAUR, MS4A6A, HLA-DQB1, IFITM3, MS4A4A, IFITM2, IFI30, NAMPT, CPVL, TYMP, TGFBI, PPA1, BCL2A1, HLA-DRB1, SMIM3, SOCS3, LMNA, TMEM176A, F13A1, FCGR3A, HLA-DRB5, DUSP2, PLIN2, HLA-DPB1, LDHA |
| **Microglia** | CH25H, ACY3, DHRS9, CCL3L1, CCL3L3, P2RY12, CCL3, RPS4Y1, CCL4L1, EGR2, TSC22D3, CCL4, SYNDIG1, PDK4, DDX3Y, SLC44A2, RIN2, CX3CR1, CCL4L2, EGR1, HNRNPH1, IPCEF1, EGR3, FOS, BHLHE41, RGS1, C12orf75, ITGAX, JUN, PLA2G7 |
